# *Campylobacter jejuni* and *Campylobacter coli* autotransporter genes exhibit lineage-associated distribution and decay

**DOI:** 10.1186/s12864-020-6704-z

**Published:** 2020-04-19

**Authors:** Jai W. Mehat, Roberto M. La Ragione, Arnoud H. M. van Vliet

**Affiliations:** Department of Pathology and Infectious Diseases, School of Veterinary Medicine, University of Surrey, Guildford, UK

**Keywords:** *Campylobacter*, *Jejuni*, *Coli*, Autotransporter proteins, Genomics, Recombination

## Abstract

**Background:**

*Campylobacter jejuni* and *Campylobacter coli* are major global causes of bacterial gastroenteritis. Whilst several individual colonisation and virulence factors have been identified, our understanding of their role in the transmission, pathogenesis and ecology of *Campylobacter* has been hampered by the genotypic and phenotypic diversity within *C. jejuni* and *C. coli.* Autotransporter proteins are a family of outer membrane or secreted proteins in Gram-negative bacteria such as *Campylobacter,* which are associated with virulence functions. In this study we have examined the distribution and predicted functionality of the previously described *capC* and the newly identified, related *capD* autotransporter gene families in *Campylobacter*.

**Results:**

Two *capC*-like autotransporter families, designated *capC* and *capD,* were identified by homology searches of genomes of the genus *Campylobacter*. Each family contained four distinct orthologs of CapC and CapD. The distribution of these autotransporter genes was determined in 5829 *C. jejuni* and 1347 *C. coli* genomes. Autotransporter genes were found as intact, complete copies and inactive formats due to premature stop codons and frameshift mutations. Presence of inactive and intact autotransporter genes was associated with *C. jejuni* and *C. coli* multi-locus sequence types, but for *capC*, inactivation was independent from the length of homopolymeric tracts in the region upstream of the *capC* gene. Inactivation of *capC* or *capD* genes appears to represent lineage-specific gene decay of autotransporter genes. Intact *capC* genes were predominantly associated with the *C. jejuni* ST-45 and *C. coli* ST-828 generalist lineages. The *capD3* gene was only found in the environmental *C. coli* Clade 3 lineage. These combined data support a scenario of inter-lineage and interspecies exchange of *capC* and subsets of *capD* autotransporters.

**Conclusions:**

In this study we have identified two novel, related autotransporter gene families in the genus *Campylobacter*, which are not uniformly present and exhibit lineage-specific associations and gene decay. The distribution and decay of the *capC* and *capD* genes exemplifies the erosion of species barriers between certain lineages of *C. jejuni* and *C. coli*, probably arising through co-habitation. This may have implications for the phenotypic variability of these two pathogens and provide opportunity for new, hybrid genotypes to emerge.

## Background

*Campylobacter jejuni* and *Campylobacter coli* are important zoonotic pathogens that are recognised as the principal causative agents of bacterial gastroenteritis [1, 2]. *C. jejuni* and *C. coli* are common commensals of poultry [3] with broiler chickens being the primary reservoir accounting for up to 80% of human infection [4]. These organisms are also common inhabitants of the gastrointestinal tract of other food producing animals such as cattle, pigs and sheep [5]. Dominant *Campylobacter* genotypes, belonging to the ST-21 clonal complex, ST-45 clonal complex and ST-828 clonal complex, exhibit a multi-host, generalist lifestyle [6–8]. By contrast, other *C. jejuni* lineages exhibit a host-adapted population structure in which certain genotypes are associated with a particular host species or ecological niche [9]. Similarly, certain lineages of *C. coli* have been linked to the swine production environment as well as the non-agricultural, environmental niche [10].

*C. jejuni* and *C. coli* show significant phenotypic diversity [11–15], and vary considerably in their ability to both adhere to and invade human intestinal epithelial cells in vitro [15]. Furthermore, *C. jejuni* genotypes vary in their infection ecology of the chicken host [16]. *C. jejuni* and *C. coli* show high mutation rates and are known to recombine with DNA obtained by natural transformation [17], a trait that drives population heterogeneity and can impact upon pathogenicity. For example, single nucleotide polymorphisms in *porA*, encoding the major outer membrane protein, have been shown to give rise to hyper-virulence in ruminants [18]. Many key surface molecules of *Campylobacter* are phase variable which may also impact upon variation in infection [19–22]. Large scale recombination within the *Campylobacter* genome, often associated with niche adaption has also been observed to impact upon infection potential [23].

Autotransporter proteins are the largest and most diverse class of secretory virulence determinants in Gram-negative bacteria [24, 25]. These surface-exposed or secreted proteins share a mechanism of export, conferred by their C-terminal β-barrel structure whilst virulence properties are conferred by their N-terminal functional or “passenger” domain [24]. We recently described the CapC autotransporter in the commonly utilised reference strains *C. jejuni* 81,116 [26] and *C. jejuni* M1 [27], which is absent in the reference isolates *C. jejuni* NCTC 11168 and *C. jejuni* 81–176 [28]. Advances in sequencing technology have resulted in the public availability of large collections of genome sequences of *C. jejuni* and *C. coli* [29], which have been used to show distinct distribution patterns of gene families involved in pathogenesis, metabolism and stress responses [23, 30–32]. Autotransporter proteins often occur in families within a bacterial species or genus [33], and the distribution of such autotransporter families in isolates from distinct backgrounds may aid our understanding of phenotypic variation in *Campylobacter* species, and shed light on host specificity and niche adaption of different *Campylobacter* genotypes.

In this study we used publicly available *Campylobacter* genome sequences to demonstrate that the CapC autotransporter of *C. jejuni* 81,116 is a representative of a larger family of *Campylobacter* autotransporters. Furthermore, we identify a related family of autotransporters, CapD, that are related to, but distinct from CapC, and have determined the distribution, genotype associations and extent of gene decay of the *capC* and *capD* genes within the genus *Campylobacter*, focusing on *C. jejuni* and *C. coli*.

## Results

### Identification of the *capC* and *capD* autotransporter families in *Campylobacter* species

**Fig. 1:**
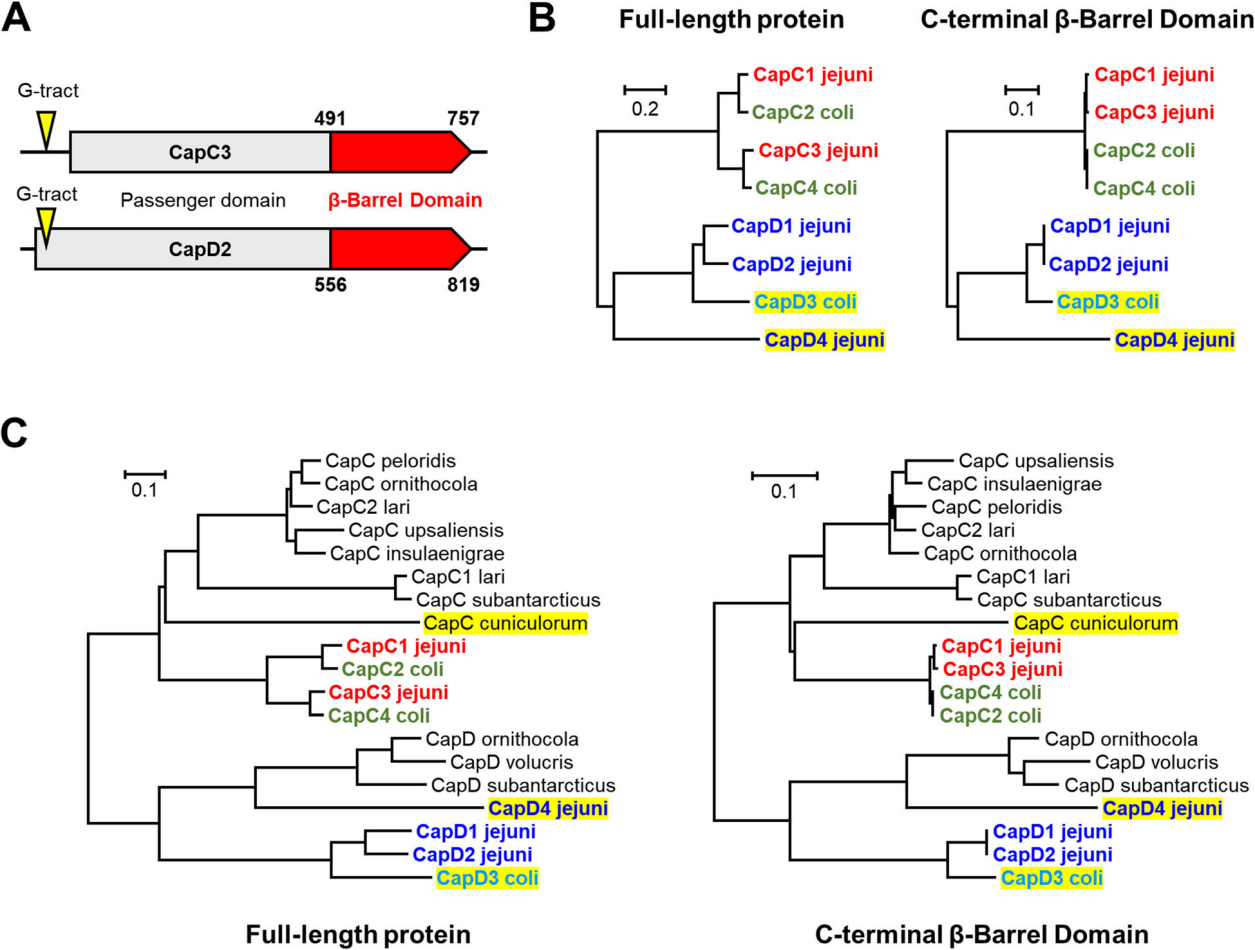
**a** Schematic representation of the alignment of *capC3* and *capD2* genes which are representative of the larger *capC* and *capD* families. The C-terminal β-barrel domain (red) between *capC* and *capD* genes is strongly conserved yet the N-terminal passenger domain sequence (grey) is highly divergent. The homopolymeric tract (denoted by yellow arrow heads) associated with *capC* autotransporters is upstream of the start codon, in the putative promoter region. The homopolymeric tract associated with *capD* autotransporters is located within the coding sequence. **b** Alignment trees generated using MEGA7 based on full length protein sequences (left) and the conserved C-terminal sequence (right) displaying the relatedness of CapC and CapD autotransporters identified in this study. Clustering of each of these two, distinct families is clear. Highlighted in yellow are autotransporter genes that lack a homopolymeric tract. **c** Alignment trees generated using MEGA7 based on full length protein sequences (left) and the conserved C-terminal sequence (right) displaying the relatedness of autotransporters belonging to the CapC and CapD families identified in a range of *Campylobacter* species. Highlighted in yellow are autotransporter genes that lack a homopolymeric tract

Initial screenings with the CapC protein sequence from *C. jejuni* 81,116 (C8J_1278) against *C. jejuni* and *C. coli* genomes from Genbank showed that there were several sequence variants present in addition to CapC in the *C. jejuni* and *C. coli* genome sequences. These autotransporter genes exhibited considerable sequence divergence in the N-terminal “passenger” domain yet share significant identity in their C-terminal domains (Fig. 1a) [25, 28]. The phylogenetic tree in Fig. 1b shows that the newly identified CapC-like autotransporters separate into two, defined clusters; one which we named CapC as it includes the originally described *capC* autotransporter described in *C. jejuni* 81,116 and *C. jejuni* M1 [28], designated *capC1*. Another cluster was named CapD and this encompasses the *capD* autotransporter family. In addition to the divergence in protein sequence, a major difference between the *capC* and *capD* autotransporter families is the location of a homopolymeric G-tract. In *capC* autotransporters, the poly-G tract is located upstream of the coding sequence in the putative promoter region whilst in the *capD* autotransporter family the poly-G tract is located in the coding sequence or is absent entirely (Fig. 1). Autotransporter genes belonging to the *capC* family were identified in *C. peloridis*, *C. ornithicola*, *C. lari*, *C. upsaliensis*, *C. subantarcticus* and *C. cuniculorum* (Fig. 1c). Autotransporter genes belonging to the *capD* family were detected in *C. ornithicola*, *C. volucris* and *C. subantarcticus* (Fig. 1c). Alignment of the complete amino acid sequences of those autotransporters as well as alignment of only the C-terminal region of each autotransporter (Fig. 1c) illustrates the division of all autotransporters detected in *Campylobacter* into the distinct *capC* and *capD* families. The position of the poly-G tract for *capC* and *capD* is conserved throughout the genus *Campylobacter* (Fig. 1c).

### Genetic characterisation of *capC* and *capD* autotransporters in *C. jejuni* and *C. coli*

In order to fully characterise the extent and distribution of autotransporter genes in *C. jejuni* and *C. coli,* each *capC* and *capD* variant was used to screen a collection of 5829 *C. jejuni* and 1347 *C. coli* genomes (Additional file 1). The *capC* and *capD* autotransporters share a degree of similarity (Fig. 1a, b, Additional file 2) in their signal peptide and C-terminal β-barrel domain, but are highly dissimilar in the N-terminal domain. Genes belonging to the CapC family were tentatively designated *capC2*, *capC3* and *capC4,* respectively, in addition to the original *capC1* gene from *C. jejuni* 81,116. A high degree of sequence similarity was observed between *capC1* and *capC2*, and *capC3* and *capC4* (Fig. 1b). Genes belonging to the CapD family were designated as *capD1*, *capD2* and *capD4* in *C. jejuni*, and *capD3* in *C. coli*. In *C. jejuni* and *C. coli*, the *capC1*-C*4* genes were all present at the same genomic position, in between the *ppk* gene (encoding a polyphosphate kinase) and the *ssrA* gene encoding a transfer-messenger RNA. These *capC* genes are mutually exclusive as they occupy the same genomic position, suggesting recombination and genotype compatibility as the major driver of heterogeneity. We did not detect any genomes containing multiple *capC* genes in their intact forms. The extended regions upstream and downstream of the *capC* locus were largely conserved between strains except for the *cj1365c* gene in *capC*-negative strains. The *capD1* and *capD2* genes are also mutually exclusive in *C. jejuni* and *C. coli* and are present between the *murA* gene, involved in peptidoglycan synthesis and *fspA2,* encoding a flagella-related protein [34]. This location is not conserved in *C. coli* Clade 3 which encodes the *capD3* gene between the *moeA* gene, involved in molybdenum metabolism [35], and a tRNA/ATPase gene. In the single genome containing *capD4*, the gene is next to an ABC transporter encoding gene and a contig end.

As the N-terminal part of autotransporters often determines specific targets or functionality, we used predictive software algorithms to investigate the CapC1-C4 and CapD1-D4 proteins. Autotransporter proteins display similarities and differences in their signal peptides, protein size and localisation (Additional file 5), which justifies their differentiation into separate families. CapC proteins have identical signal peptides and similar predicted protein sizes. However, CapC2 and CapC4 are predicted to have dual localisation sites in the outer membrane and secreted extracellularly. CapD autotransporters vary in their signal peptide composition and cleavage site as well as protein size. CapD1 and CapD2 are predicted to be secreted extracellularly, whereas CapD3 and CapD4 are predicted to localise to the outer membrane proteins. This indicates a high degree of structural conservation within the C-terminal of CapC and CapD autotransporter proteins, and a high degree of variation in the N-terminal domains, but does not provide further information on functionality of these domains.

### Lineage-specific associations of intact and inactive autotransporters

**Fig. 2:**
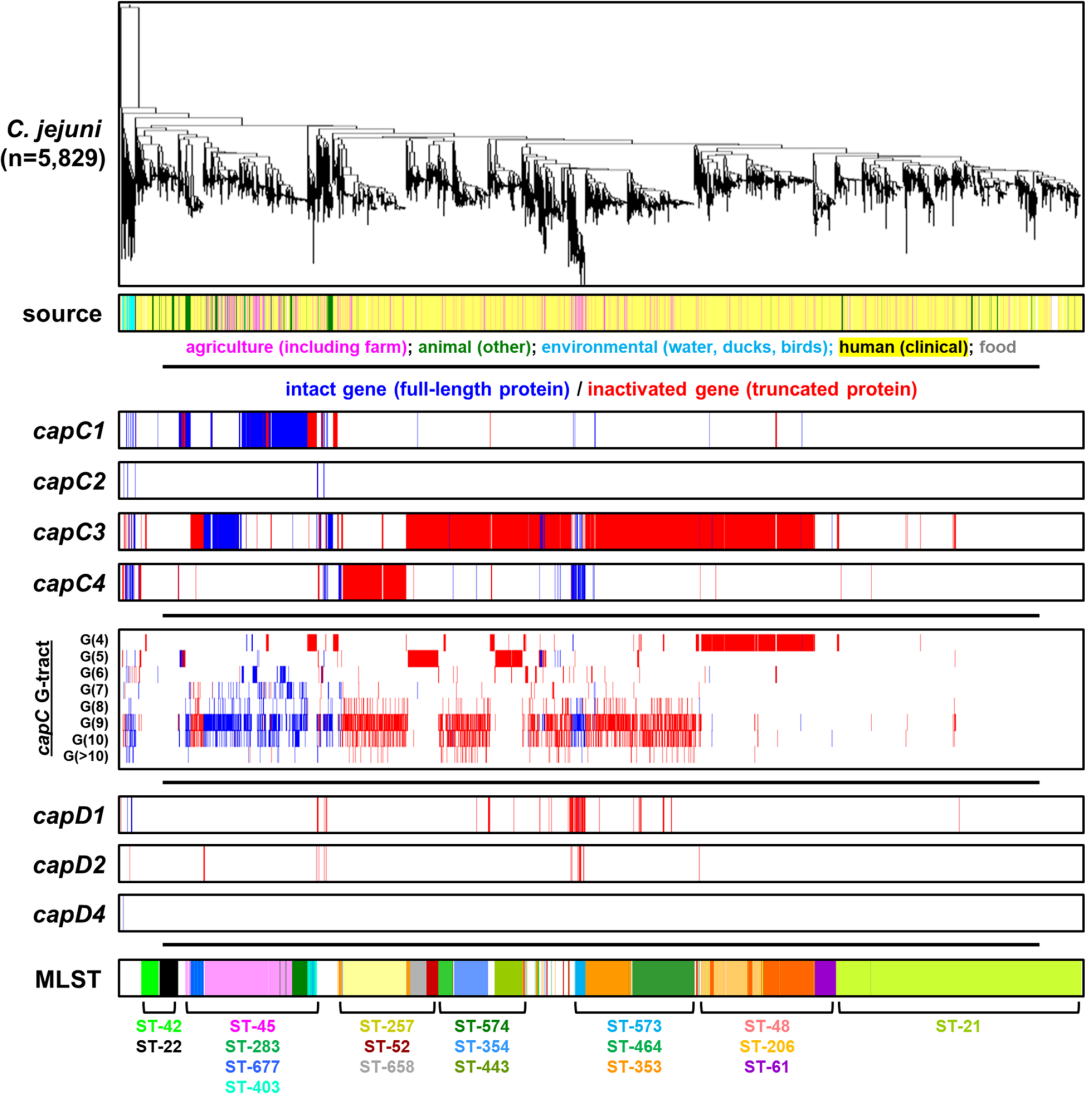
Prevalence and genotypic associations of autotransporter genes in *C. jejuni*. A total of 5829 genomes were phylogenetically clustered using Feature Frequency Profiling with a word length of 18. This clustering was depicted in a phylogenetic tree using Figtree. The first row beneath the resulting tree labelled isolation source indicates the source of isolation for each genome within the collection via colour coding with labels directly beneath this row. Rows labelled “*capC1”*, “*capC2”, “capC3”,* “*capC4”, “capD1”, “capD2”* and *“capD4”* indicate whether the corresponding genomes possesses either intact (dark blue colouring) or inactive (red colouring) formats of each of these genes. No colouring in these rows indicates the absence of a particular autotransporter gene. The box in the middle of the figure labelled “*capC* G-tract” indicates the length of the homopolymeric tract in the putative promoter region of the *capC* gene detected within a particular genome. Dark blue colouring indicates the *capC* or *capD* gene is intact whereas red colouring indicates whether the capC or capD gene is inactive. G-tract length ranges from 4 to ≥10. The final row shows the associated MLST clonal complex of the corresponding *C. jejuni* genomes

**Fig. 3:**
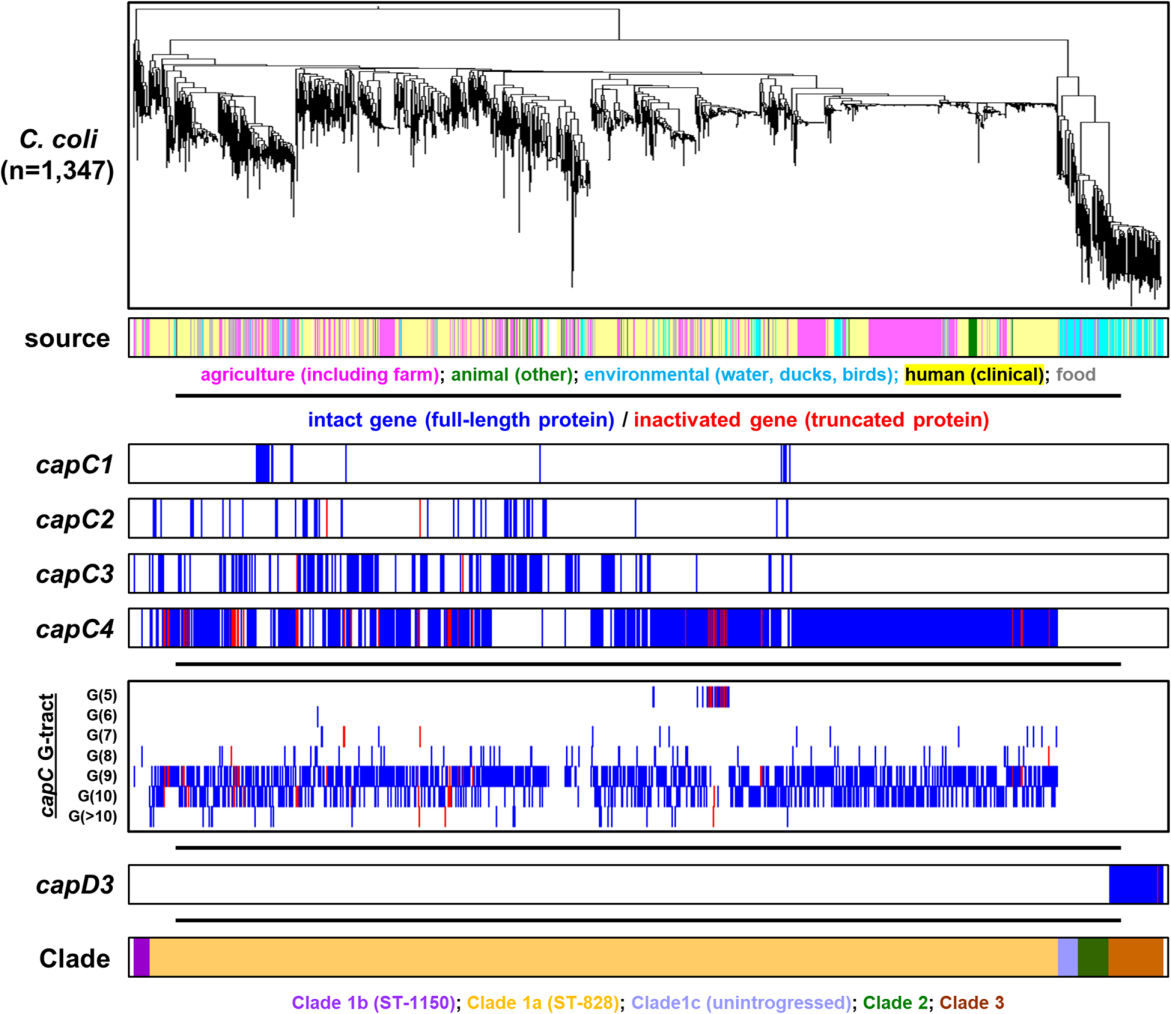
Prevalence and genotypic associations of autotransporter genes in *C. coli*. A total of 1347 genomes were phylogenetically clustered using Feature Frequency Profiling with a word length of 18. This clustering was depicted in a phylogenetic tree using Figtree. The first row beneath the resulting tree labelled isolation source indicates the source of isolation for each genome within the collection via colour coding with labels directly beneath this row. Rows labelled “*capC1”*, “*capC2”, “capC3”,* “*capC4”* and *“capD3”* indicate whether the corresponding genomes possesses either intact (dark blue colouring) or inactive (red colouring) formats of each of these genes. No colouring in these rows indicates the absence of a particular autotransporter gene. The box in the middle of the figure labelled “*capC* G-tract” indicates the length of the homopolymeric tract in the putative promoter region of the *capC* gene detected within a particular genome. Dark blue or Red colouring indicates whether the *capC* or *capD* gene is intact or inactive, respectively. G-tract length ranges from 5 to ≥10. The final row shows the associated phylogenetic clade of the corresponding *C. coli* genomes

The 7176 *C. jejuni* and *C. coli* genome sequences (Additional file 1) were screened for the presence of *capC* and *capD* genes to determine whether the genes detected are intact and therefore predicted to encode a full-length protein, or whether the genes detected are inactive and predicted not to encode a functional protein (Figs. 2 and 3, Table 1, Table 2, Additional file 1). Autotransporter genes, in both intact and inactive formats, are present in most clonal complexes in *C. jejuni* and *C. coli* although there were notable associations with specific *C. jejuni* and *C. coli* genetic backgrounds. For example, whilst there are instances of *capC1* in genomes belonging to numerous clonal complexes, it is predominantly associated with the ST-283 clonal complex and a sub-group of the ST-45 clonal complex (Fig. 2). Moreover, the distribution of intact and inactive autotransporter genes was associated with specific MLST genotypes of *C. jejuni* and *C. coli*. For instance, inactive *capC3* is highly pervasive in *C. jejuni* and is present in a wide range of MLST genotypes including the ST-658, ST-52, ST-574, ST-354, ST-443, ST-353, ST-464, ST-573 ST-61, ST-206 and ST-48 clonal complexes. However, the complete, intact gene is mostly present in the ST-45 clonal complex and the ST-573 clonal complex. Similarly, the *capC4* gene is associated with numerous clonal complexes in its complete, intact form, but is inactive in the ST-257 clonal complex (Fig. 2, Additional file 1). This apparent linkage of inactive and intact autotransporter genes with genetic background is also observed in *C. coli* which has a more defined genomic population structure. The *capC1*-C*4* autotransporters are closely associated with *C. coli* Clade1a/ST-828 and are absent from Clade 2 and 3, whereas the *capD3* autotransporter is exclusively associated with *C. coli* Clade 3.

**Table 1:** The number and proportion of genomes within major *C. jejuni* clonal complexes and *C. coli* Clades from the collection used in this study that encode intact and inactive *capC* autotransporter genes. The number and proportion of genomes that do not encode *capC* or *capD* is also shown

Clonal	Total	***capC1***				***capC2***				***capC3***				***capC4***				***capC***/***capD***	
Complex	Genomes	Intact		Inactive		Intact		Inactive		Intact		Inactive		Intact		Inactive		absent	
**ST-21**	**1500**	0	–	0	–	0	–	0	–	0	–	45	(3%)	0	–	2	(0.13%)	1452	(96.8%)
**ST-22**	**112**	0	–	0	–	0	–	0	–	0	–	0	–	0	–	0	–	112	(100%)
**ST-42**	**105**	1	(0.95%)	0	–	0	–	0	–	0	–	8	(7.61%)	0	–	0	–	96	(91.4%)
**ST-45**	**543**	309	(56.9%)	20	(3.68%)	0	–	0	–	203	(37.3%)	9	(1.65%)	0	–	0	–	2	(0.36%)
**ST-48**	**375**	2	(0.53%)	7	(1.86%)	0	–	0	–	3	(0.8%)	361	(96.2%)	0	–	0	–	2	(0.53%)
**ST-52**	**82**	0	–	0	–	0	–	0	–	0	–	82	(100%)	0	–	0	–	0	–
**ST-61**	**130**	0	–	0	–	0	–	0	–	1	(0.76%)	4	(3.07%)	0	–	0	–	125	(96.1%)
**ST-206**	**300**	0	–	0	–	0	–	0	–	0	–	297	(99%)	0	–	1	(0.33%)	2	(0.66%)
**ST-257**	**394**	0	–	0	–	0	–	0	–	0	–	19	(4.82%)	0	–	375	(95.1%)	0	–
**ST-283**	**99**	98	(98.9%)	0	–	0	–	0	–	1	(1.01%)	0	–	0	–	0	–	0	–
**ST-353**	**339**	4	(1.17%)	0	–	0	–	0	–	3	(0.88%)	311	(91.7%)	18	(5.30%)	1	(0.29%)	2	(0.58%)
**ST-354**	**214**	0	–	0	–	0	–	0	–	0	–	213	(99.5%)	1	(0.46%)	0	–	0	–
**ST-403**	**56**	0	–	55	(98.2%)	0	–	0	–	0	–	1	(1.78%)	0	–	0	–	0	–
**ST-443**	**168**	0	–	0	–	0	–	0	–	1	(0.59%)	0	–	3	(1.78%)	0	–	0	–
**ST-464**	**379**	0	–	0	–	0	–	0	–	0	–	377	(99.4%)	0	–	0	–	2	(0.52%)
**ST-573**	**61**	1	(1.63%)	0	–	0	–	0	–	14	(22.9%)	3	(4.91%)	43	(70.4%)	0	–	0	–
**ST-574**	**99**	0	–	0	–	0	–	0	–	3	(3.03%)	96	(96.9%)	0	–	0	–	0	–
**ST-658**	**110**	1	(0.90%)	0	–	0	–	0	–	0	–	108	(98.1%)	0	–	1	(0.90%)	0	–
**ST-677**	**78**	0	–	0	–	0	–	0	–	0	–	77	(98.7%)	0	–	1	(1.28%)	0	–
**None**	**434**	26	(5.99%)	5	(1.15%)	10	(2.30%)	1	(0.23%)	65	(14.9%)	222	(51.1%)	32	(7.37%)	25	(5.76%)	46	(10.5%)
**Clade1a (ST-828)**	**1189**	29	(2.43%)	0	–	60	(5.04%)	2	(0.16%)	204	(17.1%)	3	(0.25%)	787	(66.1%)	51	(4.28%)	54	(4.54%)
**Clade1b (ST-1150)**	**20**	0	–	0	–	0	–	0	–	1	(5%)	0	–	1	(5%)	0	–	18	(90%)
**Clade1c**	**26**	0	–	0	–	0	–	0	–	0	–	0	–	0	–	0	–	26	(100%)
**Clade 2**	**40**	0	–	0	–	0	–	0	–	0	–	0	–	0	–	0	–	40	(100%)
**Clade 3**	**72**	0	–	0	–	0	–	0	–	0	–	0	–	0	–	0	–	3	(4.16%)

**Table 2:** The number and proportion of genomes within major *C. jejuni* clonal complexes and *C. coli* Clades from the collection used in this study that encode intact and inactive *capD* autotransporter genes

Clonal	Total	***capD1***				***capD2***				***capD3***				***capD4***			
Complex	Genomes	Intact		Inactive		Intact		Inactive		Intact		Inactive		Intact		Inactive	
**ST-353**	**339**	0	–	1	(0.29%)	0	–	0	–					0	–	0	–
**ST-354**	**214**	0	–	2	(0.93%)	0	–	0	–					0	–	0	–
**ST-443**	**168**	0	–	1	(0.59%)	0	–	0	–					0	–	0	–
**ST-464**	**379**	0	–	21	(5.54%)	0	–	0	–					0	–	0	–
**ST-573**	**61**	1	(1.63%)	54	(88.50%)	0	–	13	(21.30%)					0	–	0	–
**ST-661**	**13**	0	–	10	(76.90%)	0	–	1	(7.69%)					0	–	0	–
**ST-692**	**12**	0	–	1	(8.33%)	0	–	0	–					0	–	0	–
**None**	**434**	4	(0.92%)	47	(10.80%)	0	–	14	(3.22%)					1	(0.23%)	0	–
**Clade 3**	**72**									68	(94.40%)	1	(1.38%)				

### Homopolymeric G-tract length does not influence intact or inactive status of *capC*

Homopolymeric guanine/cytosine tracts mediate adaptive mutations in *Campylobacter* species through slipped-strand mispairing of these repetitive sequences [21, 36]. Variation in the homopolymeric tract identified in the coding sequence of *capD* autotransporters will influence inactivation of *capD* genes, but whether the poly-G tract upstream of *capC* genes influences inactivation of the downstream gene was not known. The poly-G tract upstream of the *capC1* start codon in the *C. jejuni* 81,116 reference genome is also present at the equivalent site in *capC-C4*-positive genomes (Fig. 1a). To determine whether this homopolymeric tract influenced the observed inactivation of *capC* genes, we compared the length of poly-G tracts with the active/inactive status of the downstream autotransporter gene (Fig. 2 and Fig. 3). In *C. jejuni*, tract length ranged from G = 4 to G ≥ 10 and the most common tract length was G9 (Fig. 2, Additional file 1). *capC* autotransporters within the same clonal complex were determined to be intact at a range of poly-G tract lengths; for example, in ST-45 the complete, intact *capC1* and *capC3* are present with poly-G tract lengths of G4 to G10. Similarly, the G-tract length of inactive *capC4* in *C. jejuni* ST-257 ranges from G8 to G ≥ 10. Furthermore, in *C. coli,* intact and inactive *capC* autotransporters were present with tract lengths of G7, G8, G9 and G10. These results indicate that homopolymeric tract length does not correspond with whether *capC* autotransporter genes are intact or inactive and that intact or inactive status of *capC* autotransporters is closely associated with clonal complex (Additional files 3 and 4).

## Discussion

The autotransporter family is comprised of many important bacterial virulence factors in Gram-negative pathogens [24, 33]. These proteins consist of an N-terminal “passenger” domain which determines the effector function of the autotransporter [24], and a C-terminal β-barrel domain which facilitates insertion into the bacterial outer-membrane [25]. The CapC1 autotransporter has been shown to contribute to virulence in *C. jejuni* and the CapA autotransporter has been reported to be involved in adhesion to epithelial cells and chicken colonisation [28, 37, 38], although we do not yet know the exact mechanism by which CapC1 contributes to virulence. Bioinformatic analysis of the passenger domains of CapC1-C4 and CapD1-D4 did not result in identification of specific domains that may explain such functionality (Additional file 5).

In this study, we have described two novel autotransporter families in *Campylobacter* and report the lineage-specific distribution and decay of these autotransporter genes. Notably, we determined that *capC* autotransporters are shared between *C. jejuni* and *C. coli* lineages [39]. The *capC* and *capD* autotransporter genes are common throughout *C. jejuni* and *C. coli* in either their inactive or intact forms, except for select lineages which do not appear to encode CapC- or CapD autotransporters (Additional file 1). There is a clear, defined sub-population within ST-45 containing *capC3* rather than *capC1*. The degree of demarcation between lineages that encode certain autotransporters is exemplified by this sub-population and is evidence of strong genotype associations rather than with isolation source. Due the linkage of genotype and ecological niche observed in *Campylobacter* [9], observed associations of an autotransporter with a particular genetic lineage may cause an indirect association with an isolation source. These associations may be exaggerated considering that the collection of publicly available *Campylobacter* genomes used in this study is heavily comprised of human clinical isolates belonging to ST-21 and Clade 1a *C. coli*, which are more readily available than isolates from other sources. Human infections are commonly transmitted via poultry or ruminant sources, but for these human isolates the transmission route is not known. The high frequency with which ST-21 and Clade 1a *C. coli* isolates appear in the dataset can skew interpretations regarding the proportion of autotransporter genes encoded by *Campylobacter*. Ecological association displayed by certain genotypes does not preclude events leading to transmission of isolates to different niches. Definitive source attribution is difficult in *Campylobacter* species [40, 41], particularly with multi-host adapted lineages which display poor host specificity markers [6]. Therefore, potential associations of autotransporters with ecological niches via quantitative source attribution, are difficult to accurately infer. Ultimately, possession of *capC* and *capD* autotransporters is correlated with the genetic background of *C. jejuni* and *C. coli*.

Intact *capC* autotransporters are predominantly associated with the ST-45 and ST-283 clonal complexes in *C. jejuni* and the ST-828 (Clade 1a) clonal complexes in *C. coli*. Considering the high degree of inactive *capC* genes in other clonal complexes, the high proportion of intact, functional *capC* in ST-45 and ST-828 is striking and could be indicative of a functional role for these autotransporters in colonisation of the agricultural niche or in the multi-host lifestyle exhibited by these lineages. However, ST-21 is also a generalist lineage that is prevalent within the agricultural niche, yet isolates from this clonal complex do not contain the *capC* autotransporter gene whilst thriving in these environments [42]. Rather, *C. jejuni* ST-21 often contains the *capA*/*B* autotransporter genes, which may mitigate for the absence of CapC or CapD autotransporters [28, 37]. Previous studies have demonstrated that *C. jejuni* isolates from generalist lineages readily recombine with each other in vitro*,* yet despite a considerable degree of niche overlap, the ST-45 and ST-21 lineages do not show any evidence of recombination with each other in nature [7]. Therefore, the ecological barrier that segregates these lineages may also restrict *capC* autotransporter genes to ST-45 and ST-828.

We have identified shared *capC1*-C*4* autotransporter genes between *C. jejuni* lineages and introgressed *C. coli* ST-828 (Figs. 2, 3). In *C. jejuni*, the CapC autotransporter family is restricted to select genotypes in either its intact or inactive form. However, *C. coli* ST-828 encodes predominantly intact *capC1-*C*4* autotransporters with no discernible association of each *capC* allele with sub-population structure of ST-828. Considering the similarity between *capC* autotransporter genes (Fig. 1) as well as the upstream and downstream genes, this observed incidence is consistent with interspecies sharing of *capC* autotransporters between *C. coli* Clade1a/ST-828 and multiple *C. jejuni* lineages, probably via a shared niche. Recombination between *C. jejuni* and *C. coli* ST-828 has been demonstrated previously by the accumulation of *C. jejuni* alleles by *C. coli* [39, 43, 44]*.*

Both *capC* and *capD* have homopolymeric G-tracts associated with the genes, but their respective position is distinct. The *capC* genes have poly-G tract upstream of the *capC* start codon, whereas the *capD* genes have a poly-G tract in the open reading frame or do not have a poly-G tract at all. Our analysis shows that the length of this poly-G tract, whilst variable, does not correlate with the inactive/intact status of the *capC* autotransporters and therefore does not influence inactivation of these genes (Figs. 2, 3). Coupled with the association of intact and inactivated formats with specific clonal complexes, we propose that inactivation of these genes is linked with *Campylobacter* genotype rather than homopolymeric tract length. Exceptions to this are the inactive *capC3* genes in the ST-48, ST-206 and ST-61 clonal complexes which predominantly possess a G-tract of 4 consecutive nucleotides and those in ST-443, ST-52 and ST-658 which possess G-tracts of 5 nucleotides. The *capC3* gene in these lineages all display highly similar patterns of inactivation (Additional files 3 and 4) and are decayed to such an extent as to make reversion to intact status by addition or deletion of a nucleotides upstream of the coding sequence impossible. The uniform G-tract length in these clonal complexes is likely the result of gene decay of the entire locus including the intergenic regions due to lack of maintenance pressure. It is therefore likely that a progressive process of pseudogene formation is responsible for degradation of autotransporter genes in specific lineages rather than phase variation mediated by poly-G tracts. Pseudogenisation of autotransporters suggests a functional redundancy of these genes in certain lineages, leading to inactivation once their respective functions are no longer required within a specialised niche [45, 46]. This “adaptive loss” scenario has been observed in *C. jejuni* previously and is a proposed consequence of niche differentiation [45]. Conversely, this would suggest a possible environmental pressure selecting for the maintenance of intact *capC* and *capD* in *C. jejuni* ST-45 and ST-283 and *C. coli* ST-828 and for *capD3* in *C. coli* Clade 3. Given the location of the poly-G tract, it is conceivable that strand-slippage may impact upon the expression of the *capC* genes [26, 28]. Furthermore, given the widespread sharing of *capC* autotransporters, it is possible that the intergenic regions upstream and downstream are also shared by inter-lineage and inter-species recombination making evaluation of the impact of homopolymeric tract length very difficult.

## Conclusions

In this study we report on two novel, related autotransporter families in the genus *Campylobacter* and show that *capC* and *capD* autotransporter genes display specific distribution patterns of intact and inactive genes associated with MLST clonal complexes. This widespread, lineage-specific inactivation of *capC* and *capD* genes in *Campylobacter* likely represents gene decay as a consequence of functional redundancy, host/niche adaption or a lack of environmental selection towards maintenance of intact genes, especially in *C. jejuni*. The select presence of autotransporters highlights that *Campylobacter* virulence mechanisms vary between strains and genetic backgrounds and that accessory gene distribution and decay is an important consideration when evaluating *Campylobacter* phenotypic variability. This contrasts with *capC* genes being exchanged between *C. jejuni* and *C. coli*, presumably via a shared environment and recombination. Furthermore, this pattern of genetic exchange highlights the erosion of intrinsic recombination barriers between these species arising through co-habitation. Further studies are required to fully examine interspecies recombination of *capC* autotransporters, and whether the barriers that prevent recombination of these autotransporters, thus restricting them to certain lineages, are ecological or essential in nature. Other virulence determinants and metabolic genes should also be examined to more accurately define genotype associations and the extent of exchange of genetic material between *C. jejuni* and *C. coli*.

## Methods

### *Campylobacter* genomes used in this study

A collection of 7176 complete and draft *Campylobacter* genome sequences (5829 *C. jejuni*, 1347 *C. coli*) were used in this study and obtained from PubMLST (http://pubmlst.org/campylobacter) and Genbank. These genomes are listed in Additional file 1, with PubMLST ID, Genbank accession number, isolate source category, MLST sequence type, clonal complex, *capC1–4* and *capD1–4* status and G-tract length *capC* genes included where available. For *C. coli*, the clades 1a (ST-828), clade 1b (ST-1150), clade 1c (non-introgressed), clade 2 and clade 3 [30, 39] were also determined. The assembly quality of genome assemblies was evaluated using Quast V 4.6.3 [47] and poor quality assemblies were excluded, based on aberrant genome size (< 1.5 Mbp or > 2.0 Mbp), low N_50_ (< 25 kbp), high L_50_ (> 25), and high number of Ns per 100 kb (> 50).

### Determination of the prevalence of intact and inactive autotransporters in *C. jejuni* and *C. coli*

Genome sequences were screened for the presence of the *capC1–4* and *capD1–4* genes by using Abricate version 0.8 (https://github.com/tseemann/abricate) and BLAST+ version 2.9.0 (NCBI). All genomes in the collection were annotated using Prokka [48], and these annotations were screened for complete and truncated versions of the CapC1–4 and CapD1–4 proteins using BioEdit version 7.25 [49]. The G-tract length of the *capC1–4* promoters was determined after querying the genome sequences with the promoter of the *capC1* gene of *C. jejuni* 81,116 (C8J_1278). Phylogenetic trees were created for *C. jejuni* and *C. coli* genomes using Feature Frequency Profiling with a word length of 18 [50], as used previously for earlier collections of *C. jejuni* and *C. coli* genome sequences [31, 32]. Colour-coding of intact and inactive gene encoding isolates within a phylogenetic tree generated using Figtree allowed associations of autotransporters with genotypes to be visualised.

### Bioinformatic tools for comparison of CapC and CapD autotransporter families

SignalP 5.0 (http://www.cbs.dtu.dk/services/SignalP/), CELLO V2.5 (http://cello.life.nctu.edu.tw/), NCBI Conserved Domain Database (http://www.ncbi.nlm.nih.gov/Structure/cdd/wrpsb.cgi), Phyre2 (http://www.sbg.bio.ic.ac.uk/phyre2/html/page.cgi?id=index) and Protein Molecular Weight Calculator (https://www.bioinformatics.org/sms/prot_mw.html) were used to identify signal peptides, conserved domains, autotransporter protein size and subcellular localisation of CapC and CapD autotransporters.

## Supplementary information

**Additional file 1. **Table of *C. jejuni* and *C. coli* genomes used in this study showing Genbank accession numbers, *capC*/*capD* presence or absence and intact or inactivation status.

**Additional file 2. **A) Alignment of amino acid sequences of CapC1/2/3/4 and CapD1/2/3/4 variants in *C. jejuni* and *C. coli.* B) Alignment of amino acid sequence of CapC and CapD variants in *Campylobacter* species.

**Additional file 3. **Figure displaying the fragmentation patterns of inactive *capC3* and *capC4* genes. The figure shows the various frameshifts (FS) and point mutations that result in inactive genes; these mutations are associated with the clonal complex in which the inactive *capC*3/*capC4* is present.

**Additional file 4.** A) Table showing the number of genomes in major clonal complexes that encode frameshift (FS) or premature stop mutations in CapC3/CapC4 at specific amino acid residues. B) Table showing the percentage of genomes in major clonal complexes that encode frameshift (FS) or premature stop mutations in CapC3/CapC4 at specific amino acid residues.

**Additional file 5.** Table showing summary of results from comparison of autotransporter amino acid sequences using searching for conserved domains, signal sequences, protein size and predicted localisation sites.

## Data Availability

All data generated or analysed during this study are included in this published article [and its Additional information files] and is publicly available from http://pubmlst.org/campylobacter and https://www.ncbi.nlm.nih.gov/genome. Genbank Accession Numbers: *C. jejuni* CapC1: WP_002866779.1; *C. coli* CapC2: WP_052793243.1; *C. jejuni* CapC3: WP_022552386.1; *C. coli* CapC4: WP_023362112.1; *C. jejuni* CapD1: WP_126232584.1; *C. jejuni* CapD2: WP_126216674.1; *C. coli* CapD3: WP_020974791.1; *C. jejuni* CapD4: WP_070298870.1; *C. lari* CapC1: WP_114640428.1; *C. lari* CapC2: WP_074691797.1; *C. peloridis* CapC: WP_044598937.1; *C. ornithocola* CapC: WP_066008681.1; *C. insulaenigrae* CapC: WP_039650305.1; *C. cuniculorum* CapC: ARJ56787.1; *C. volucris* CapD: WP_039665304.1; *C. upsaliensis* CapC: translated as frameshifted protein from NZ_UFUZ01000001.1; *C. subantarcticus* CapC: WP_039664182.1 (N-terminus) and WP_082018437.1 (C-terminus); *C. ornithocola* CapD: OCX42345.1 (C-terminal part, N-terminal part translated from genome sequence LXSU01000139.1); *C. subantarcticus* CapD: N-terminus translated from genome sequence, MPB98625.1 (middle part), MPB98624.1 (C-terminus).

## Abbreviations

*C. jejuni*: *Campylobacter jejuni*
*C. coli*: *Campylobacter coli*
ST: Sequence Type
MLST: Multi-Locus Sequence Type
tRNA: transfer-ribonucleic acid
ATPase: adenosine triphosphate hydrolase
Poly-G: homopolymeric guanine tract
bp: base pairs
