# Supplementary material for: Campylobacter jejuni and Campylobacter coli autotransporter genes exhibit lineage-associated distribution and decay
Source: BMC Genomics. 2020 Apr 19;21:314. doi: 10.1186/s12864-020-6704-z (PMC7168839; doi:10.1186/s12864-020-6704-z)
Supplement: Supplementary file 2 — Additional file 2. A) Alignment of amino acid sequences of CapC1/2/3/4 and CapD1/2/3/4 variants in C. jejuni and C. coli. B) Alignment of amino acid sequence of CapC and CapD variants in Campylobacter species. [file 12864_2020_6704_MOESM2_ESM.pdf]

A. Alignment of amino acid sequences of CapC and CapD variants in *C. jejuni* and *C. coli*

```
CapC1_jejuni      MKKNASSKILLSLGVATLLYS---GAFAAEITFNGDSDLDKYFDINEKDNVATFKNENYK
CapC2_coli        MKKNASSKILLSLGVATLLYS---GAFAAEITFNSDSDLNTHFDINEKDNVATFKNENYK
CapC3_jejuni      MKKNASSKILLSLGVATLLYS---SAFAQEINLTQSSDVGNYFEENGKD-INLKNPDKYK
CapC4_coli        MKKNASSKILLSLGVATLLYS---GAFAQEINLAGSSDIGKYFEENGKD-INLKNPDQYK
CapD1_jejuni      --MIYHNKLVLSAIAASFIVSAGGGLYAQDISNDTAAKL-----TQDKDNKSHYTYEFSK
CapD2_jejuni      --MRHHKSIMLSAIAASFIVAHGGGLYAQDISNDT--KL-----TQNKDNKSHYTYEFNT
CapD3_coli        --MKYHKKIMLSAIAASFLVAQ--GLYSANIIDNKL--------IQDSNDKSKYIYEFST
CapD4_jejuni      MRNSHNKKVTLSLGVAKVLLSA--TIFASFANAATTPDATSI--QLDDSKLTHLGNGDYQ
                        ..: **  .*..: :     ::                   ..

CapC1_jejuni      -------NKQDVTFN--ISTSAFDDAPEDTKI--------NIDLGNNS---LTLKNQMDY
CapC2_coli        -------N-QDLTFK--INTLAFDDAPEDAKI--------NIDLGNNS---LTLENTRNS
CapC3_jejuni      -------G-QDLNIK--MGVWDLPNDDYDSADYRL-----NIDIGKNNT--LSFTHNNG-
CapC4_coli        -------G-QDLSIK--MGIGDLPSDGYDSADYRF-----NIDIGKNNT--LSFTHNNN-
CapD1_jejuni      -------G-DIVKTE--LGKTKLTND--ENRIKHL-----TIKAGTGAT-ALAFEGNTG-
CapD2_jejuni      -------G-DVVKTE--LGKTKLSGE--DPIK-HL-----TIKAGKDQSGGLEFTGTTS-
CapD3_coli        -------N-EIVKDD--LGSTKLTED--ENRIKYL-----DIRAGNKQTGGLTFQGM---
CapD4_jejuni      FVISKPMG-EIIYINNTLGENEVLDEKGSAIKTVLPMVPKNITINAKGDTVIINGGQLMN
                         . : :  .  :.   .     .            *  .      :

CapC1_jejuni      QGKTAALVKNFNVDAKDFKTTDIGLSY----FNAGIINANF-------------------
CapC2_coli        GGETAALVRNFNVDAKDFKTTDIGLSY----FNAGIINANF-------------------
CapC3_jejuni      --QNPAYVTNLNATAKEVKTTDIVLQA----FAPSVINGNL-------------------
CapC4_coli        --QEPAYVTNLNATAKEVKTTDIVLQA----FAPSVINGNL-------------------
CapD1_jejuni      ---TRASIGGDISNGYSFDLTADSVSF----KGTTNSGDTT-------------------
CapD2_jejuni      ---QKASLG----DGYSFNLTADSLKF----TGDAIGSANI-------------------
CapD3_coli        ------YLGGNLNNGFSFNLTADKVQ-----FNKDDKYLNA-------------------
CapD4_jejuni      GDRTWAGIGGAESN-YNFTINADTVKLIGTEYNDDDLNDNFGVQVLPDGSLWQPDYNNPY
                         :        ..  .   :.             .

CapC1_jejuni      ----------TMEGSGKDFDLG-NIDKNKASSLLIFNGSRENTNDTVNGSLTVNGDFSTT
CapC2_coli        ----------TMVGSGEDFDLD-NIDKNKASSLLIFNGSRENTNDTVNGSLTVNGDFSAT
CapC3_jejuni      ----------TMTSSGAGTITE---DEKKGSGIILYNGAVEGKS--ANGSLTINGNFTAD
CapC4_coli        ----------TMTSSGKEAITQ---DEEKGSGIILYNGAVEGKS--ANGSLTINGNFTAD
CapD1_jejuni      ----------INVGSGHSTINAKNGVTLEKTSIDLANGNYFNKN--FNGSLTINGDLKLT
CapD2_jejuni      ----------IATNGASTIDAK-NGVELNKGHIQVGNGTFFNQS--FNGSLTINGDLKLT
CapD3_coli        ----------IYVGSGDSIITAKGGVELNDTFINIATGDYFNHS--YNGSLTINGNLKLV
CapD4_jejuni      NSLLDTYHDNTFMNVFKTGVIN-GNLETTAASVAIVNGNLFGRN--YNGNLTVNGNLISN
                               .                  : : .*   . .   **.**:**::

CapC1_jejuni      NSAIVSMKSDTFKVNGTATLKEAGLGFLSQSYSNLDVNDFIALRA-KDIKTDTLNEDTNA
CapC2_coli        NSAIVSMKSDTFKVNGTATIEKSGLGFLSQSYSNLDANDFIVLRA-KDIKTDKLNDETNA
CapC3_jejuni      KTLFATYGNF-VKVNGTANLTNSNFGLMKRSYTDLEANNVVMVQA-KDFNENILKANNNA
CapC4_coli        KTLFATYGNF-VKVNGTANLTNSNFGLMKRSYTDLEANNVVIVQA-KDFNKDILEEKSNN
CapD1_jejuni      DSSVINNGQAGFNVNGKVTANNTEFVAGVGDLNRVTNNGFFIMSATKGFENGIEKDKSFT
CapD2_jejuni      DSSVTNFGQANFNVNGKVSAQNTTFETEALDLSRVTSNGFYIMSATKGFENAASSDKSFT
CapD3_coli        DSSILNQGGAKFHVNGKVIAFDTQFSVSVDDFDRVTNNGIVIMTASEGFES-LDKDKTFT
CapD4_jejuni      DTKFTFFGNHQFNVSGTAIIRDSEFSLVNFS-NPVTENGIYLLSSGKGLNKDITETNTVR
                  .: .       .:*.*..   .: :     .   :  *..  : : :.::    .  ..

CapC1_jejuni      -------GAL-----ILKTASSYINENLLNGDDYAAY--LDVT-DDKKYGG--AFVDYKL
CapC2_coli        -------GAL-----ILKSGSSYIDESLLNGDDYVAS--LDVTAEDNKYGG--VFVDYKL
CapC3_jejuni      -------GAL-----LLKFASDYISTDVQGKDPLEAGTIIDISDEDKYGDGEKGLVDYKL
CapC4_coli        -----NAGAL-----LLKFASDYISTNVQGKDPLEAGTVLDIT--DDKYSG--GLVDYKL
CapD1_jejuni      DVSSHNTGALVFHNSLVNISSNLLDSKYAGGFGTITEANFATIAPERDLS---ALYDTRL
CapD2_jejuni      DVSSGNKGALVFHNPLVNISTNLMNQNYAGGVGTITD--FDQIAPTTDLS---SLYATEL
CapD3_coli        DIDSGNFGVIYVHNKLVNISSNLMDSKYAKDIGTITEADFKKIAPTHDLSDRDSLYETEL
CapD4_jejuni      NVLLIPDGKL----------LGLKGDKFAEYEGELTEIILDKDN---------KLFTNKL
                         * :           .  . .        :   :              :   .*

CapC1_jejuni      SLKNCGGDKCLV----------------------------------INGGATAAAKNLTN
CapC2_coli        SLKNCGGDKCLV----------------------------------INGGATAAAKKLTN
CapC3_jejuni      SVQNCGGNKCLV----------------------------------INGGATAAAKDKLV
CapC4_coli        SVQNCGGNKCLV----------------------------------VNGGATAAAKDKLV
CapD1_jejuni      DIK---GNSLYVVGDLKGFDANTIKNFKTAK---DWENAFKTLSTNIQTQFTAQKTDLEK
CapD2_jejuni      VQK---GNSLYVNG-----------NFATIK---NGRVDFDALNAAIKTEFTKQKTELEK
```

```
CapD3_coli        VQQ---GNNLYVSG-----------NFDKAKFYTDGRLDFDKLNTALKKEYAAQKSDLEI
CapD4_jejuni      VVS---GNTLYV-----------------------------------KNSLTAEAKKMSI
                    .   *:.  *                                   :   :   ..

CapC1_jejuni      QIAVD-------------LEAITRII-DGLDNEQ------AKKALQEQKTELEKLQQEAM
CapC2_coli        QIAVD-------------LEAITRII-DGLDNEQ------AKETLKKQQAEIQTMLEEAK
CapC3_jejuni      QLQVD-------------IDAIDKLLKNEFDSSQDEEWTKAKEALEKQKTELEQLKQEAE
CapC4_coli        QLQVD-------------IDTINKLLENEFDSSQDEEWTKAKETLKKQQAELQTMLEEAK
CapD1_jejuni      LYKYDSSSKAESGIFKNNRDTVKNKI-DEYNKEKTGIIAKSQQAVDTTKAALDKANQEGK
CapD2_jejuni      LYKVD--GKKESGTFFTNKGLIEKEI-TKYNTEKTGTIATSQADVDSKKTALDKANQEGK
CapD3_coli        LYKNN--AGNESGFFLDKRQRVDAEI-KAHAIE----ISSSEAKVSSAKLTLDETKKGND
CapD4_jejuni      GEFLK----------ETRTQILDQLI--AHFTEQYNDSITGSGIQPRAATTVGATAQATQ
                      .                :   :      .       ..         :    :

CapC1_jejuni      QNGGKIDDEKYIDLVNK---------NSNLNLSANDKASILVLRSITEQLGSI-G---AD
CapC2_coli        KNGGKIDDEKYIDLVNK---------NLNLNLSANDKASILALRSITEQLGSI-G---AD
CapC3_jejuni      KNGGKIDDEKYIDLVNK---------NSNLNLSANDKASILALRSITEQLGSI-G---AD
CapC4_coli        KNGGKIDDEKYIDLVNK---------NSNLNLSANDKASILALRSITEQLGSI-G---AD
CapD1_jejuni      QETIKTAQKNYDTAVKQLENDKKTLSELNNRLAEYNALIEDIKNKTANLNKKIND---TN
CapD2_jejuni      QEEIKKAQEAYDTAVKKLEDDKKTLSELNNRLAEYNTLIEDIKNKTANLNDKINN---AK
CapD3_coli        FKRIEEAQKAYDDAKKKLDSDKKILDELKNRLAEYDRLIEEVKNKTADLDKKITGFADGG
CapD4_jejuni      YKKIIEQLTQYKNDI-----------EANKKVVSYGKNVDNILASLKNSNREV-A---AS
                   :        *               : : .:   .        .  :   .:

CapC1_jejuni      LASREGVKLAL------------------------QIKKDTDNTGKSVSNFNSASSAVNT
CapC2_coli        LASREGVKLAL------------------------DIKKDTDNTGKSVSNLNSASSAVNT
CapC3_jejuni      LASREGVKLAL------------------------QIKKDTDNTGKSVSNLNSASSAVNT
CapC4_coli        LASREGVKLAL------------------------DIKKDTDNTGKSVSNLNSASSAVNT
CapD1_jejuni      FNIAAGQKGQIFASLATSSVGGKLAGTADYIFANGKVINDVERAAQSNAGNSALNAPIG-
CapD2_jejuni      FDIRAGQKGQIFASLAESNVGGKLAGTADYIFANGKVINDVERAAQSNAGNSALNAPIG-
CapD3_coli        SSVQMGQKGQIFASLAESNVGGKVSATAGYIFSNDKAIDDIEKAGRSNAGNSALNSPVN-
CapD4_jejuni      SALLNAIKGNV------------------------NQAKTIEASAKESADNSNRQGAIQ-
                       . *  :                        .  .  : :.:. :. .  ...:

CapC1_jejuni      TMNISNDVSIGSRVAMLNNPFGTY-ASKMNGLKFAA---LDSDMRPSY-VNEYTNSVWAN
CapC2_coli        TMNISNDVSIGSRVAMLNNPFGTY-ASKMNGLKFAA---LDSDMRPSY-VNEYTNSVWAN
CapC3_jejuni      TMNISNDVSIGSRVAMLNNPFGTY-ASKMNGLKFAA---LDSDMRPSY-VNEYTNSVWAN
CapC4_coli        TMNISNDVSIGSRVAMLNNPFGTY-ASKMNGLKFAA---LDSDMRPSY-VNEYTNSVWAN
CapD1_jejuni      AINMSNDMAISNRLAKFSNPYSTVNVASLEGLKFAAGNGIASDSQYAYGARSYDNNVWAN
CapD2_jejuni      AINMSNDMAISNRLAKFSNPYSTVNVASLEGLKFAAGNGIASDSQYAYGARSYDNNVWAN
CapD3_coli        AINISNEMTISNRLAKFSNPYSTIKLASLAWEKFAAGEGIASDSTFSYGARAYENNIWAN
CapD4_jejuni      VINLANEMAISNRMAQLSNST----------------------------TNDLNNGFWAN
                  .:*::*:::*..*:* :.*.                             ..   *..***

CapC1_jejuni      AFGGANIIDGDSGAMYGATVGVDKQANDNVLWGAYFTYANAKIKDNNLEQKSDNFQLGMY
CapC2_coli        AFGGANIIDGDSGAMYGATVGVDKQANDDVLWGAYFTYANAKIKDNNLEQKSDNFQLGMY
CapC3_jejuni      AFGGANIIDGDSGAMYGATVGVDKQANDDVLWGTYFTYANAKIKDNNLEQKSDNFQLGMY
CapC4_coli        AFGGANIIDGDSGAMYGATVGVDKQANDDVLWGAYFTYANAKIKDNNLEQKSDNFQLGMY
CapD1_jejuni      VIGGANIIDSKSGALYGVSVGYDRLVGEDTILGAYLTYADSKIKTSMVNQESDNLQLGLY
CapD2_jejuni      VIGGANIIDSKSGALYGVSVGYDRLVGEDTILGAYLTYADSKIKTSMVNQESDNLQLGLY
CapD3_coli        VIGGANIIDSNSGSLYGVSVGYDRLIGEETILGAYLTYANSETKTSTIEQKSDNLQLGLY
CapD4_jejuni      GFGGGNFL-GDNESVYGMSVGFDRKVGDDIIVGGYLTYADSTLTNNSISQDSDNLQLGLY
                   :**.*:: ... ::** :** *:  .:: : * *:***::  . . :.*.***:***:*

CapC1_jejuni      STINIAPQWELNLKAYAQVSPTKQDNVQVDGAYNSDYTSKFLGLSANAGRVFDLSDNTLF
CapC2_coli        STINVAPQWELNLKAYAQVSPTKQDNVQIDGAYNSDYTSKFLGLSANAGRVFDFSDNTLF
CapC3_jejuni      STINIAPQWELNLKAYAQVSPTKQDNVQVDGAYNSDYTSKFLGLSANAGRVFDLSDNTLF
CapC4_coli        STINVAPQWELNLKAYAQVSPTKQDNVQIDGAYNSDYTSKFLGLSANAGRVFDFSDNTLF
CapD1_jejuni      SRT-LYGNSEFDFKGYAQFGWTDQDRFIAGTVNSSDFTRKFLGASGTYGYVFDM-GNDFY
CapD2_jejuni      SRT-LYGNSEFDFKGYAQFGWTDQDRFIAGTVNSSDFTRKFLGASGTYGYVFDM-GNDFY
CapD3_coli        SRT-LYNNSEFDFKTYAQFGWTDQNRLIAQTINSSDFTRKFLGASGSYGYVFDI-GNDLY
CapD4_jejuni      SRI-ATGSHEFDIKGYTQFSFADQNRMINDSVQTSDFTQTFLGLSGAYGYVFNL-NNGFA
                  *      . *:::* *:*.. :.*:..      .**:* .*** *.  * **:: .* :

CapC1_jejuni      IKPFAGVNYYFSYTPSHTENGAIAKDIDSMKNNSVSVEVGAEFRKYMNENSYIFVTPKIE
CapC2_coli        IKPFAGVNYYFSYTPSHTENGAIAKDIDSMKNNSVSVEVGAEFRKYMNENSYIFVTPKIE
CapC3_jejuni      IKPFAGVNYYFSYTPSHTENGAIAKDIDSMKNNSVSVEVGAEFRKYMNENSYIFVTPKIE
CapC4_coli        IKPFAGVNYYFSYTPSHTENGAIAKDIDSMKNNSVSVEVGAEFRKYMNENSYIFVTPKIE
CapD1_jejuni      IKPLAGLNLYYSFTPDYNENGAYAQHVQSQSSFDTSIEAGAEFRKYLSKESYIYATPKIE
CapD2_jejuni      IKPLAGLNLYYSFTPDYNENGAYAQHVQSQSSFDTSIEAGAEFRKYLSKESYIYATPKIE
CapD3_coli        IKPLAGLNLYYSLTPSYNENGPYAQHVRSQSNFDASIEAGAEFRKYLSKESYIYATPKIE
```

```
CapD4_jejuni      IKPLAGLNLYYSHTPDYTEEGLWAQHVNSMDSFAFSGDLGVEFRKFFDGGSYIYITPKIE
                  ***:**:* *:* **.:.*:*  *:.: * ..   * : *.****::.  ***: *****

CapC1_jejuni      QFVINSGDDYTANLAVNNAFFTSVEANNKKKTYGQIIVGGNVDFTNQLSMNLGFGAKQIL
CapC2_coli        QFVINSGDDYTANLAVNNAFFTSVEANNKKKTYGQIIVGGNVDFTNQLSMNLGFGAKQIL
CapC3_jejuni      QFVINSGDDYTANLAVNNAFFTSVEANNKKKTYGQIIVGGNVDFTNQLSMNLGFGAKQIL
CapC4_coli        QFVINSGDDYTANLAVNNAFFTSVEANNKKKTYGQIIVGGNVDFTNQLSMNLGFGAKQIL
CapD1_jejuni      QYIITSGDDYTARFLGSPTSFT-INGSDKKKTYGSFIVGGDVNIKNQWAFTFSAGVKHLL
CapD2_jejuni      QYIITSGDDYTARFLGSPTSFT-INGSDKKKTYGSFIVGGDVNIKNQWAFTFSAGVKHLL
CapD3_coli        QYIITSGDHYTARFLGSPTSFT-INGSDKKKTYGSLIVGGDVNIKNAWAFTFSAGIKHLL
CapD4_jejuni      QFFVVDGDVFRSGFTGSNLKYS-IHGDESNKTYGQIILGSDISVSKNFSINVSAGIKQIL
                  *:.: .** : : :  .   :: :...:.:****.:*:*.::...:  ::... * *::*

CapC1_jejuni      AGKVDNKNETYLSGQVGLKYKF
CapC2_coli        AGKVDNKNETYLSGQVGLKYKF
CapC3_jejuni      AGKVDNKNETYLSGQVGLKYKF
CapC4_coli        AGKVDNKNETYLSGQVGLKYKF
CapD1_jejuni      SGKVNDESETYLSGNIGLKYQF
CapD2_jejuni      SGKVNDESETYLSGNIGLKYQF
CapD3_coli        SGKANNESETYLSGNIGLKYQF
CapD4_jejuni      GNKDSNTDETYVSGNLGATYKF
                  ..* .: .***:**::* .*:*
```

B. Alignment of amino acid sequence of CapC and CapD variants in *Campylobacter* species

```
CapC1_jejuni             ---MKKN-ASSKILLSLGVATLLYSGA---FAAEITFN-GDSDLDKYFDINEKD-----N
CapC3_jejuni             ---MKKN-ASSKILLSLGVATLLYSSA---FAQEINLT-QSSDVGNYFE-ENGK-----D
CapD1_jejuni             ---MI---YHNKLVLSAIAASFIVSAGGGLYAQDISND-------TAAKLTQDK-----D
CapD3_coli               ---MK---YHKKIMLSAIAASFLVAQG---LYSANIID---------NKLIQDS-----N
CapC4_coli               ---MKKN-ASSKILLSLGVATLLYSGA---FAQEINLA-GSSDIGKYFE-ENGK-----D
CapC2_coli               ---MKKN-ASSKILLSLGVATLLYSGA---FAAEITFN-SDSDLNTHFDINEKD-----N
CapC_upsaliensis         ---MKLSYHASKVLVGVSISALLSSVA---LVQDISIN--TGNAKNYFDTNDGQ-----H
CapC1_lari               ---MKLSYHTSKVLMGVSISALLSSAT---LAQEINYI----NLSDYFNYKDGI------
CapC2_lari               ---MKLSYYTSKVLMSVSIGALLSSAA---LTQEITITDGDGSMEQHFETNDGQ-----H
CapC_subantarcticus      ---MKLSYYTSKVLMGVSISALLSGAA---LAQEINYI----NLSDFFKYKDGI------
CapD_subantarcticus      ---MK---NSRKFFLSACVATMVASSA---HAVTKKKG-GGGSFSDTNLKDINK-----N
CapC_peloridis           ---MKLSYHTSKVLMGVSISALLSSAV---LAQEITIN--DSTMNQYFETSDGR-----N
CapC_ornithocola         ---MKLSYHTSKVLMGVSISALLSSAA---LAQEITIY--DSNMDQYFETSDGR-----N
CapD_ornithocola         ---MRN---SRKIFLSTCVATMVASSA---HAVI---GGGGISFTDSNLKDENK-----N
CapD_volucris            ---MKN--LNRKIFLSACVATMVASSA---HA----VTGGGNPFSDTNLKDENK-----N
CapC_insulaenigrae       ---MKLSYFASKVLIGFSIGALLSSNA---LASKITIKDGDGSMEKHFETNDGQ-----H
CapC_cuniculorum         MIKTKLSSTTCKVLLGMSLA--LSASN---LYAAKNVD-SNATIKENFE-KKGN-----S
CapD4_jejuni             ---MRNS-HNKKVTLSLGVAKVLLSAT---IFASFANA-ATTPDATSIQLDDSKLTHLGN
CapD2_jejuni             ---MR---HHKSIMLSAIAASFIVAHGGGLYAQDISND---------TKLTQNK-----D
                                    .. :.   .  : .

CapC1_jejuni             VATFKNENYKNKQDVTF-------------NISTSAFDDA-PEDTK--------INIDLG
CapC3_jejuni             INLKNPDKYKG-QDLNI-------------KMGVWDLPNDDYDSADYR------LNIDIG
CapD1_jejuni             NKSHYTYEFSK-GDIVKT------------ELGKTKLTND-ENRIKH-------LTIKAG
CapD3_coli               DKSKYIYEFST-NEIVKD------------DLGSTKLTED-ENRIKY-------LDIRAG
CapC4_coli               INLKNPDQYKG-QDLSI-------------KMGIGDLPSDGYDSADYR------FNIDIG
CapC2_coli               VATFKNENYKN-QDLTF-------------KINTLAFDDA-PEDAK--------INIDLG
CapC_upsaliensis         FALKK--EHAN-SDLNI-------------KMDSMDLSND-IAKDGYEDRA-T-VGIDLG
CapC1_lari               WSLD-----SK-ENIEL-------------KITPSDIQSY-YGGENH-DKP-I-KEFNIK
CapC2_lari               FTLKN--DYTN-GNLTI-------------NINDTDLPNS-IAKEGYEDLA-T-VNINLG
CapC_subantarcticus      WSLD-----SK-ENIEL-------------KITPSDIQSY-YGGENH-DKP-I-KEFNIK
CapD_subantarcticus      GNYSFVVEKDT-GSI---------------YNISSVIGES-FDGENNKHIK-H-LNIDAA
CapC_peloridis           FNLKK--DYTN-GDLTL-------------NLFHTDLANS-IVNDKYKDLS-R-VNINLN
CapC_ornithocola         FNLKK--DHTN-SDLTL-------------NLYHTDLANA-IAKDEYKDLS-R-VNINLG
CapD_ornithocola         GNYSFIVDKDT-GSI---------------YNISSVIGSS-FDGKTNKFIN-H-LNIDAA
CapD_volucris            GNYSFIDKNTG-STYNI----------------TSIIGST-FDGKTNKYIN-H-LNIDAA
CapC_insulaenigrae       FALKK--EHTN-SDLTI-------------EINDTDLPNS-IAKEGYEDLA-T-VSIDLG
CapC_cuniculorum         FSYKGTDYDIALGNISTQGWDDDDTKAPTEETSLFDEKGT-TKFKNFNAKDEK-ISIDAG
CapD4_jejuni             GDYQFVISKPM-GEIIYINN----------TLGENEVLDE-KGSAIKTVLPMVPKNITIN
CapD2_jejuni             NKSHYTYEFNT-GDVVKT------------ELGKTKLSGE--DPIKH-------LTIKAG
                                                                                 :

CapC1_jejuni             NNS-LTLKNQMDY---QGKTAALVKNF-NVDAKDFKTTDIGL------------------
CapC3_jejuni             KNNTLSFTHNN-----GQNPAY-VTNL-NATAKEVKTTDIVL------------------
CapD1_jejuni             TGA-TALAFEGN----TGTRASIGGDISNGYSFDLTADSVSF------------------
CapD3_coli               NKQTGGLTFQGM---------YLGGNLNNGFSFNLTADKVQF------------------
CapC4_coli               KNNTLSFTHNNN-----QEPAY-VTNL-NATAKEVKTTDIVL------------------
CapC2_coli               NNS-LTLENTRNS---GGETAALVRNF-NVDAKDFKTTDIGL------------------
CapC_upsaliensis         AHN-LSFENGNS----GNKASY-VTNY-TITAKKQKLLMVIF------------------
CapC1_lari               TNGILTIGDGDNWIDVGSTSTN-EAKY-DLYSVNLEAKEIIL------------------
CapC2_lari               SNE-LTIKNISH----GDISTY-VTNY-TINAKKTEAIDVIF------------------
CapC_subantarcticus      TSGILTIGDEDNWIDVGSTSTN-EAKY-DLYSVNLEAKEIIL------------------
CapD_subantarcticus      KST-LKVGANDQD---AKIGHYHDGKKDKFYSFDIKAKEIIF------------------
CapC_peloridis           SNN-LTFKNT------SESASY-VTNY-TINAKKTEATDVIF------------------
CapC_ornithocola         SNN-LTFKNT------SEGANY-VTNY-TINAKKTEATDVIF------------------
CapD_ornithocola         KST-FNVGANGQD---AEIGHYFDGEKDKFYSFNIKAKEIIF------------------
CapD_volucris            KST-FNISVKDKD---AEIGHYFDGEKDKFYSFNIKAKEIIF------------------
CapC_insulaenigrae       SND-LTIKNISS----GNLSTA-VSNY-TITANKTEATNVIF------------------
CapC_cuniculorum         EKK-LTLSTTGA----DTLNNFTLTST-EASADAIKATGINF------------------
CapD4_jejuni             AKGDTVIINGGQLMN-GDRTWAGIGGAESNYNFTINADTVKLIGTEYNDDDLNDNFGVQV
CapD2_jejuni             KDQSGGLEFTGT--------TSQKASLGDGYSFNLTADSLKF------------------
                               .                                : :

CapC1_jejuni             ---------SYFN-AGIINANFTMEGS----GKDFDLGNI---DKNKASSLLIFNGSREN
CapC3_jejuni             ---------QAFA-PSVINGNLTMTSS----GAGTITE-----DEKKGSGIILYNGAVEG
CapD1_jejuni             ---------KGTT--NSGDTTINVGSG----HSTINAKNG---VTLEKTSIDLANGNYFN
```

```
CapD3_coli                ---------NKDD--KYLNAIYVGSGD-----SIITAKGG---VELNDTFINIATGDYFN
CapC4_coli                ---------QAFA-PSVINGNLTMTSS----GKEAITQ-----DEEKGSGIILYNGAVEG
CapC2_coli                ---------SYFN-AGIINANFTMVGS----GEDFDLDNI---DKNKASSLLIFNGSREN
CapC_upsaliensis          ---------QSPDGKSIVNEDFNIKGS-----SNPTEDAL---DDIKASAILIGDGHGV-
CapC1_lari                ---------NSDRATLEANKAFNIKGNVTLNGSSPITGDNINDWELDRPSLDVWNGYGE-
CapC2_lari                ---------QSTNGKSIVNGDFSIKGS-----SSPTEGVL---DDIKSSAILIGDGHGL-
CapC_subantarcticus       ---------NKDQASLKANKAFNIKGNLTLNGSSPITNDNIHNEELDRPGLYVWNGYGE-
CapD_subantarcticus       ---------DGIN-------SALEVGN-----TGVIEGNL---HIINGANLSVSNGTLGS
CapC_peloridis            ---------QSLDGKSIVNGDFSIKGS-----SNPVEGVL---DDVKSSAILIADGHGL-
CapC_ornithocola          ---------QSLDGKSIVNGDFSIKGS-----SNPVEGVL---DDVKSSAILIADGHGL-
CapD_ornithocola          ---------DGTN-NTLGQETSLNVGN-----TGVIEGDL---ALRNGADLFISNGTLGS
CapD_volucris             ---------NGAS-DGMGKETSLNVGN-----TGVIEGNL---ALLNSADLFISNGTLGS
CapC_insulaenigrae        ---------QSMNGKSIVNGDFSIKGS-----SEPTEGVF---DYMKSSAIFIADGHGL-
CapC_cuniculorum          ---------RATSGSSVFNGNTSLSGG-----------------------TLIVDG----
CapD4_jejuni              LPDGSLWQPDYNNPYNSLLDTYHDNTFMNVFKTGVINGNL----ETTAASVAIVNGNLFG
CapD2_jejuni              ------------TGDAIGSANIIATNG----ASTIDAKNG---VELNKGHIQVGNGTFFN
                                                                      :  *

CapC1_jejuni              TNDTVNGSLTVNGDFSTTNSAIVSMKSD----TFKVNGTATLKEAGLG-FLSQSYSNLDV
CapC3_jejuni              KS--ANGSLTINGNFTADKTLFATYGN-----FVKVNGTANLTNSNFG-LMKRSYTDLEA
CapD1_jejuni              KN--FNGSLTINGDLKLTDSSVINNGQA----GFNVNGKVTANNTEFV-AGVGDLNRVTN
CapD3_coli                HS--YNGSLTINGNLKLVDSSILNQGGA----KFHVNGKVIAFDTQFS-VSVDDFDRVTN
CapC4_coli                KS--ANGSLTINGNFTADKTLFATYGN-----FVKVNGTANLTNSNFG-LMKRSYTDLEA
CapC2_coli                TNDTVNGSLTVNGDFSATNSAIVSMKSD----TFKVNGTATIEKSGLG-FLSQSYSNLDA
CapC_upsaliensis          -----QGSLDVNGNFTADKSTLFTIGGNKSQNHIQVNGKANITNSNFS-IGTTSFGDLAL
CapC1_lari                ----KDGYMNIQGNLNVNNSFIGIYDANKKGGLISVDGDVNIKDSAIG-ISTNSVSNLGI
CapC2_lari                -----QGSLEVNGNFTADQSTLFTIGGNKSQNHIQVNGKANITNSNFS-IGTTSFGGLAL
CapC_subantarcticus       ----KDGYMSIQGNLNVNNSFIGIFDANKKGGLISVDGDVNIKDSAIG-ISTNSVSNLGI
CapD_subantarcticus       QS--AYGSLLVKGDFAAEDAKFDFYGNH----NFHVTGKANIINSTFK----TSNAPVSE
CapC_peloridis            -----QGSLEVNGNFTADKSTLFTIGGNKSQNHIQVNGKANITNSNFS-IGTTSFGDLAL
CapC_ornithocola          -----QGSLEVNGNFTADQSTLFTIGGNKSQNHIQVNGKANITNSNFS-IGTTSFGDLAL
CapD_ornithocola          QS--ANGSLLVKGNFSATNANLDFYGNH----SFHITGKANITNSTFS----ATNAPVSE
CapD_volucris             QS--ANGSLLVKGDFLATDANLDFYGNH----NFHVTGTAFIRNSNFS----ATNAPISE
CapC_insulaenigrae        -----QGSLEINGNFTADKSILATIGGNKSQNHIQVNGKANITNSNFS-IGTTSFGDLAL
CapC_cuniculorum          ------GSINVNGALALANTKLAFNADRLS--AIQVKGNANLGTLATGSVLLSSKSIQSF
CapD4_jejuni              RN--YNGNLTVNGNLISNDTKFTFFGNH----QFNVSGTAIIRDSEFS--LVNFSNPVTE
CapD2_jejuni              QS--FNGSLTINGDLKLTDSSVTNFGQA----NFNVNGKVSAQNTTFE-TEALDLSRVTS
                                * : ::* :   .: .           . : * .

CapC1_jejuni              NDFIALRAKDI------KTDTL---NEDTNAGALI------LKTASSYIN--ENLLNGDD
CapC3_jejuni              NNVVMVQAKD-------FNENIL--KANNNAGALL------LKFASDYIS--TDVQGKDP
CapD1_jejuni              NGFFIMSATKG------FENGI---EKDKSFTDVSSHNTGALVFHNSLVNI-SSNLLDSK
CapD3_coli                NGIVIMTASEG------F-ESL---DKDKTFTDIDSGNFGVIYVHNKLVNI-SSNLMDSK
CapC4_coli                NNVVIVQAKD-------FNKDILEEKSNNNAGALL------LKFASDYIS--TNVQGKDP
CapC2_coli                NDFIVLRAKDI------KTDKL---NDETNAGALI------LKSGSSYID--ESLLNGDD
CapC_upsaliensis          NNYVFMSASGG------FNENI---TSSNKASANI------SKSFESIVGM-SNKDLGLD
CapC1_lari                NNYVAIKTTGN------FNQDI----DKNMVTALY------TKDITSMLET-SNLLPKNV
CapC2_lari                NNYVFMSASEG------FNEDI---TDSNKASANI------SKSFESIVGM-SNKDLGLD
CapC_subantarcticus       NNYVAIKTTGN------FNQDI----DKNIVTALY------TKDITSMLET-SNLLPKNV
CapD_subantarcticus       NGIFLLSANGG------FNKNI---TTSNTAGVYK--EFYLDKDENIFISILTGKTPGYI
CapC_peloridis            NNYVFMSASEG------FNEDI---TNSNKASANI------SKNFESIVGM-SSKDLGLD
CapC_ornithocola          NNYVFMSASEG------FNEDI---TSSNKASANM------SKNFESIVGM-SSKDLGLS
CapD_ornithocola          NGIFLLSADGG------FNKNL---TTSNTAGVYT------IISTADALGL-SAEEAKKY
CapD_volucris             NGIFLLSADKG------FNKDI---ELTNTAGVYT------AISTAQALGL-SAEEAKKY
CapC_insulaenigrae        NNYVFMSASEG------FNKDI---TSSNKASANI------SKSLESIVGM-STKDLGLD
CapC_cuniculorum          NNAVLVNVTGQDEKGVPFTLQVTGLDTANVVGAKL------QVDDRTYITD-NVNLNTLS
CapD4_jejuni              NGIYLLSSGKG------LNKDI---TETNTVRNVL------LIPDGKLLG-----LKGDK
CapD2_jejuni              NGFYIMSATKG------FENAA---SSDKSFTDVSSGNKGALVFHNPLVNI-STNLMNQN
                          *.   :                      .                   :

CapC1_jejuni              YAA-------------------------YLDVT-DDKKYGG---AFVDYKLSLKNC---
CapC3_jejuni              LEAGT-----------------------IIDIS-DEDKYGDGEKGLVDYKLSVQNC---
CapD1_jejuni              YAGGFGTITEANFATIAP----------ERDLS-----------ALYDTRLDIKGN---
CapD3_coli                YAKDIGTITEADFKKIAP----------THDLSDRD--------SLYETELVQQGN---
CapC4_coli                LEAGT-----------------------VLDIT-D-DKYSG---GLVDYKLSVQNC---
CapC2_coli                YVA-------------------------SLDVTAEDNKYGG---VFVDYKLSLKNC---
CapC_upsaliensis          YEVVR-----------------------AMAVK-----------DFVDYKLSTKDN---
CapC1_lari                FFE-------------------------ATDLA-----------KFTDYKLSVSND---
CapC2_lari                YEVVR-----------------------AMDVK-----------DFVDYKLSTKDN---
CapC_subantarcticus       FFE-------------------------DTDLA-----------QFTDYKLSVSND---
CapD_subantarcticus       AAEGLVPSEG------------------TIEIEI----------EGLNYKLATIGN---
```

```
CapC_peloridis          YEVVR------------------------AIDVK-----------DFVKYELSTKDN---
CapC_ornithocola        YEVVR------------------------AMDVK-----------DFVDYELSTKDN---
CapD_ornithocola        NESFD------------------------SIKVNI----------KDVNHEIKVVGN---
CapD_volucris           EENID------------------------SIKVDI----------KGVDHKIKVVGN---
CapC_insulaenigrae      YEDTK------------------------AMDVK-----------EFVDYKLSTKDN---
CapC_cuniculorum        GVAGKGATGGVKEGDRVSFADYVNKNFSNVVDLG-----------NLVEYTLNTNETKDP
CapD4_jejuni            FAEYEGELTEI------------------ILDKDN----------KLFTNKLVVSGN---
CapD2_jejuni            YAGGVGTITDFDQIAP-------------TTDLS-----------SLYATELVQKGN---
                                                                   :

CapC1_jejuni            -GGDKCLVING--------------------GATAAAKNLTNQI----------------
CapC3_jejuni            -GGNKCLVING--------------------GATAAAKDKLVQL----------------
CapD1_jejuni            -----SLYVVGDLKGFDANTIKNFKTAK---DWENAFKTLSTNI----------------
CapD3_coli              -----NLYVSG-----------NFDKAKFYTDGRLDFDKLNTAL----------------
CapC4_coli              -GGNKCLVVNG--------------------GATAAAKDKLVQL----------------
CapC2_coli              -GGDKCLVING--------------------GATAAAKKLTNQI----------------
CapC_upsaliensis        -----KLLING--------------------GANENVLDNKKVL----------------
CapC1_lari              ---GKSLLISG--------------------GANKNVRDLAMIL----------------
CapC2_lari              -----KLLISG--------------------GANENVNNNKMIL----------------
CapC_subantarcticus     ---GKSLLISG--------------------GANENVRDLSKIL----------------
CapD_subantarcticus     -----SLYLQ---------------------ADTSAWKDYKPS-----------------
CapC_peloridis          -----KLLISG--------------------GANKNVNNNKMIL----------------
CapC_ornithocola        -----KLLISG--------------------GANENVNNNKMVL----------------
CapD_ornithocola        -----SLYISANVDTK----------------AWNNNIKTNGLAK----------------
CapD_volucris           -----SLYISAQVDTK----------------SWQNSIKTDGLAK----------------
CapC_insulaenigrae      -----KLLISG--------------------GANKNVLDNKKVL----------------
CapC_cuniculorum        VKAATQLRISG--------------------GASTNALDSKNLVAIIQGTLDRYINDAGN
CapD4_jejuni            -----TLYVKN--------------------SLTAEAKKMSIGE----------------
CapD2_jejuni            -----SLYVNG-----------NFATIK---NGRVDFDALNAAI----------------
                              * :

CapC1_jejuni            -----------AVD----------L------------------EAITRII----------
CapC3_jejuni            -----------QVD----------I------------------DAIDKLL----------
CapD1_jejuni            -----------QTQFTAQKTDLEKLYKYDSSSKAESGIFKNNRDTVKNKIDEYNKEKTGI
CapD3_coli              -----------KKEYAAQKSDLEILYKNN--AGNESGFFLDKRQRVDAEIKAHAIE----
CapC4_coli              -----------QVD----------I------------------DTINKLL----------
CapC2_coli              -----------AVD----------L------------------EAITRII----------
CapC_upsaliensis        -----------ESD-------KKYL------------------EFVKQDL----------
CapC1_lari              -----------ESEIDIRKEVLDTF------------------ENIKKNIKYDEEYNKNS
CapC2_lari              -----------ESD-------KKYL------------------ELIKTDL----------
CapC_subantarcticus     -----------KSEIDIRKEALDTF------------------EGIKKDI----EYNKNS
CapD_subantarcticus     -----------EISKNLIIAKKEVL------------------TQLNNYL----------
CapC_peloridis          -----------ESD-------KKYL------------------ELIKTDL----------
CapC_ornithocola        -----------ESD-------KKYL------------------ELIKIDL----------
CapD_ornithocola        -----------SLKEAQKLALQSMI------------------DEYTRLM----------
CapD_volucris           -----------SLREAQKLALQSMI------------------EEYTKLM----------
CapC_insulaenigrae      -----------ESD-------KKYL------------------ELIKKDL----------
CapC_cuniculorum        TGLLKSFADDNKIEFDLTKTDEAKM------------------NEVEKAAKAIQAQRAAL
CapD4_jejuni            -----------FLKETRTQILDQLI------------------AHFTEQY----------
CapD2_jejuni            -----------KTEFTKQKTELEKLYKVD--GKKESGTFFTNKGLIEKEITKYNTEKTGT
                                                :

CapC1_jejuni            ----------DGL---DNEQA--KKALQEQKTELEKLQQEAMQNGGKID-----DEKYID
CapC3_jejuni            ----KNEFDSSQD--EEWTKA--KEALEKQKTELEQLKQEAEKNGGKID-----DEKYID
CapD1_jejuni            IAKSQQAVDTTKAALDKANQEGKQETIKTAQKNYDTAVKQLENDKKTLSELNNRLAEYNA
CapD3_coli              ISSSEAKVSSAKLTLDETKKGNDFKRIEEAQKAYDDAKKKLDSDKKILDELKNRLAEYDR
CapC4_coli              ----ENEFDSSQD--EEWTKA--KETLKKQQAELQTMLEEAKKNGGKID-----DEKYID
CapC2_coli              ----------DGL---DNEQA--KETLKKQQAEIQTMLEEAKKNGGKID-----DEKYID
CapC_upsaliensis        ----ENDKNYGDK--AKIEEA--IAKLDKQIQQIDEMIASA--SSGAIS-----NDSYVK
CapC1_lari              YQKPGYIGDDAML--EAIAQA--EKELQEQIKKLEQQIKDIEANGGKPD-----GSDLVE
CapC2_lari              ----EDAKDDEGVNIEKIDEA--IAKIDEQIKQIQDMISNA---GDKIS-----DDDYIK
CapC_subantarcticus     YQKPGYIGDDAML--EAIAQA--EKELQEQIQKLEQQIQDIKDNGGKFD-----GGDLVE
CapD_subantarcticus     -------RTEARL---PIPLN--QNQQEDPNAELKKIIKKIDAEIDRL------------
CapC_peloridis          ----EDAKDDEGVNKEKIDEA--IAKIEEQIKQIQEMISNA--GDGEIS-----NDDYIK
CapC_ornithocola        ----EDAKDDEGANKEKIDEA--IAKIDEQVKQIQDMIDSA--GSGEIS-----NDDYIK
CapD_ornithocola        -------SEAPGI--QPKTDA--KQDFTK--ADYQQIIRDLTGKMNAID-----------
CapD_volucris           -------NEAPGV--EPKAAN--KQEFTK--ADYKQIIKDLTGKMNAIN-----------
CapC_insulaenigrae      ----KKEEENGNK--DKIDEA--IAKLDKQIQQIDEMIKNT--SGGIIS-----NDDYIK
CapC_cuniculorum        ANITAGKYTENRVVNGRNTPF--EVTLLADRAAYEALSKNNAAGVDKAALKYTRNDVFVQ
CapD4_jejuni            ----NDSITGSGI--QPRAAT--TVGATAQATQYKKIIEQLTQYKNDIE-----------
CapD2_jejuni            IATSQADVDSKKTALDKANQEGKQEEIKKAQEAYDTAVKKLEDDKKTLSELNNRLAEYNT
```

```
                                                           .    .

CapC1_jejuni             LVNKN------------------------------SNLNLSANDKAS----------ILV
CapC3_jejuni             LVNKN------------------------------SNLNLSANDKAS----------ILA
CapD1_jejuni             LIEDI------------------------------KNKTANLNKKIN---DTNFNIAAGQ
CapD3_coli               LIEEV------------------------------KNKTADLDKKITGFADGGSSVQMGQ
CapC4_coli               LVNKN------------------------------SNLNLSANDKAS----------ILA
CapC2_coli               LVNKN------------------------------LNLNLSANDKAS----------ILA
CapC_upsaliensis         ---ND------------------------------SSVSASNKNFVS-------------
CapC1_lari               ---NM------------------------------KDISLENKNLAV-------------
CapC2_lari               ---ND------------------------------SSVSASNKDFVS-------------
CapC_subantarcticus      ---NM------------------------------KDVSLENKNLAV-------------
CapD_subantarcticus      -----------------------------------NKSNTAFNSQID-------------
CapC_peloridis           ---ND------------------------------SSVSASNKDFVS-------------
CapC_ornithocola         ---ND------------------------------SSVSVSNKDFVS-------------
CapD_ornithocola         ----V------------------------------NDKSPVFNSQID-------------
CapD_volucris            ----I------------------------------DSKSPVFNSQID-------------
CapC_insulaenigrae       ---ND------------------------------SSVSASNKNFVS-------------
CapC_cuniculorum         NIGNIQRGNQAIVARYLDGLEMSLRQRTNNAGQTYNNADLHTQDVGN----------VAR
CapD4_jejuni             ----A------------------------------NKKVVSYGKNVD-------------
CapD2_jejuni             LIEDI------------------------------KNKTANLNDKIN---NAKFDIRAGQ
                                                             .      .

CapC1_jejuni             LRSITE----QL--GSIGADLASREGVKLALQ------IKKDTDNTGKSVSNFNSASSAV
CapC3_jejuni             LRSITE----QL--GSIGADLASREGVKLALQ------IKKDTDNTGKSVSNLNSASSAV
CapD1_jejuni             KGQIFA----SLATSSVGGKLAGTADYIFANGK-----VINDVERAAQSNAG-NSALNAP
CapD3_coli               KGQIFA----SLAESNVGGKVSATAGYIFSNDK-----AIDDIEKAGRSNAG-NSALNSP
CapC4_coli               LRSITE----QL--GSIGADLASREGVKLALD------IKKDTDNTGKSVSNLNSASSAV
CapC2_coli               LRSITE----QL--GSIGADLASREGVKLALD------IKKDTDNTGKSVSNLNSASSAV
CapC_upsaliensis         --KILD----GL---ALGKDFNAIGSIKFDKVGEQ---VANDIKASAKSISNVNQASSGI
CapC1_lari               --NMLN----SILDSKLSND--AIAALTLDTTGKNLNTLTTNTKASAKAIVN-NAQNSSV
CapC2_lari               --KILD----GL---KIGGDLNAIGSIKFDKVGEQ---VANDIKDSAKSISNVNQASSGI
CapC_subantarcticus      --NMLN----SILDSKLSND--AVAALTLDTTGKNLNTLTTNTKASAKAIVN-NAQNSSV
CapD_subantarcticus      --AVLA----SL--LRSNREQAAGSALTDAMVGNAQ--QAKNVVNSARESAN-NSNRQGA
CapC_peloridis           --KILD----GL---KLGGDFNAIGSIKFDKAGEQ---VANDIKDSAKSISNVNQASSGI
CapC_ornithocola         --KILD----GL---ALGKDFNAIGSIKFDKVGEQ---VANDIKDSAKSISNINQASSGI
CapD_ornithocola         --AVLG----SI--LTSNRQQAAGSALTDAMIGNTQ--QAKNVVNSARESAN-NSNRQGA
CapD_volucris            --AVLG----SL--LTSNRQQAAGSALTDAMIGKAQ--QAQNVINSARESAN-NSNRQGA
CapC_insulaenigrae       --KILD----GL---TLGKDFNAIGNIKFDKVGEQ---VANDIKDSAKSISNVNQASSGI
CapC_cuniculorum         QGDILDKDGNKIGTYDNGATLDAIARIKLENLGNT---LANEFRESAKSIANTNSAVSTV
CapD4_jejuni             --NILA----SL--KNSNREVAASSALLNAIKGNVN--QAKTIEASAKESAD-NSNRQGA
CapD2_jejuni             KGQIFA----SLAESNVGGKLAGTADYIFANGK-----VINDVERAAQSNAG-NSALNAP
                            :       :     .    .                      :.:   . *   .

CapC1_jejuni             NTTMNISNDVSIGSRVAMLNNPFGTYA--SKMNGLKFAA---LDSDMRPSYVN-EYTNSV
CapC3_jejuni             NTTMNISNDVSIGSRVAMLNNPFGTYA--SKMNGLKFAA---LDSDMRPSYVN-EYTNSV
CapD1_jejuni             IGAINMSNDMAISNRLAKFSNPYSTVN-VASLEGLKFAAGNGIASDSQYAYGARSYDNNV
CapD3_coli               VNAINISNEMTISNRLAKFSNPYSTIK-LASLAWEKFAAGEGIASDSTFSYGARAYENNI
CapC4_coli               NTTMNISNDVSIGSRVAMLNNPFGTYA--SKMNGLKFAA---LDSDMRPSYVN-EYTNSV
CapC2_coli               NTTMNISNDVSIGSRVAMLNNPFGTYA--SKMNGLKFAA---LDSDMRPSYVN-EYTNSV
CapC_upsaliensis         NSTINVSNDVSIGSRVAMLNNPYGNYA--MKLSQIRFAA-----NDYMGTYLD-NYKNSI
CapC1_lari               NSSIGVANDLAIGTRVAKLSNPYQDKALVEKFATTHIAA---LASDVYNYYGNSSFNNSF
CapC2_lari               NSTINVSNDVSIGSRVAMLNNPYGNYA--TKLSQIRFAT-----NDYRGNYVD-NYNNSI
CapC_subantarcticus      NSSIGVANDLAIGTRIAKLSNPYQDKALVEKFATTHIAA---LASDVYNYYGNSSFNNSF
CapD_subantarcticus      IQVINLANEMAISTRMLQDRN---------------------------------QCENSV
CapC_peloridis           NSTINVSNDVSIGSRVAMLNNPYGNYA--TKLSQIRFAA-----NDYMGNYVD-NYKNSI
CapC_ornithocola         NSTINVSNDISIGSRVAMLNNPYGNYA--MKLSQIRFAA-----NDYRGNYVD-NYKNSI
CapD_ornithocola         IQVINLANEMAISTRMMQDRN---------------------------------QGENSV
CapD_volucris            IQVINLANEMAISTRMIQSRN---------------------------------QGENSI
CapC_insulaenigrae       NSTINVSNDVSIGSRVAMLNNPYGNYA--TKLSQIRFAT-----NDYGGNYVD-NYNNSI
CapC_cuniculorum         NSVINTANDVAIGSRIAMLNNPYGSYA--SKMSELKFAA---VASDMGANYVD-SYSNGI
CapD4_jejuni             IQVINLANEMAISNRMAQLSNSTTN-----------------------------DLNNGF
CapD2_jejuni             IGAINMSNDMAISNRLAKFSNPYSTVN-VASLEGLKFAAGNGIASDSQYAYGARSYDNNV
                            :. :*:::*..*:    *                                    *..

CapC1_jejuni             WANAFGGANIIDGDSGAMYGATVGVDKQANDNVLWGAYFTYANAKIKDNNLEQKSDNFQL
CapC3_jejuni             WANAFGGANIIDGDSGAMYGATVGVDKQANDDVLWGTYFTYANAKIKDNNLEQKSDNFQL
CapD1_jejuni             WANVIGGANIIDSKSGALYGVSVGYDRLVGEDTILGAYLTYADSKIKTSMVNQESDNLQL
CapD3_coli               WANVIGGANIIDSNSGSLYGVSVGYDRLIGEETILGAYLTYANSETKTSTIEQKSDNLQL
CapC4_coli               WANAFGGANIIDGDSGAMYGATVGVDKQANDDVLWGAYFTYANAKIKDNNLEQKSDNFQL
CapC2_coli               WANAFGGANIIDGDSGAMYGATVGVDKQANDDVLWGAYFTYANAKIKDNNLEQKSDNFQL
```

```
CapC_upsaliensis        WGNVIGGTNIIDGDSGTLYGATIGMDRKINDEAIIGAYFTYANAEIKDNLLTQKSDNFQI
CapC1_lari              WGNVFGGANIIDGDSGALYGFTLGADRKINDNALLGFYFTYADSTIKDGVMEQKSDNYQF
CapC2_lari              WGNVIGGANIIDGDSGALYGSTIGMDRKINDDVIIGAYFTYANAKIKDNLLTQKSDNFQL
CapC_subantarcticus     WGNVFGGANIIDGDSGALYGFTLGADRKINDSALLGFYFTYADSTIKDGIMEQKSDNYQF
CapD_subantarcticus     WSNAFGGANMIGSENDFVYGITLGIDRQFTDAVFFGAYLTYADSKLNYNSISQDADNLQF
CapC_peloridis          WANVIGGANIIDGDSGALYGATIGMDRKINDETIIGAYFTYANAKIKDNLLTQKSDNFQF
CapC_ornithocola        WGNIIGGANIIDGDSGALYGATIGMDRKVNDDVIIGAYFTYANAEIKDNLLTQKSDNFQF
CapD_ornithocola        WSNAFGGANMIGSESDSVYGITLGIDRQFSDSVLFGAYLTYADSKLNYNSISQDADNLQF
CapD_volucris           WSNAFGGANMIGSENDSIYGITLGIDRQFSDSILFGTYLTYADSKLSHNSINQDADNLQF
CapC_insulaenigrae      WGNIIGGANIIDGDSGALYGATIGMDRKINDDVIIGAYFTYANAKIKDNLLTQKSDNFQF
CapC_cuniculorum        WANVFGGANIIDGDTGGLYGLSLGFDKQATDNVLIGGYFTYAYAEIKDNSLSQESNNFQL
CapD4_jejuni            WANGFGGGNFL-GDNESVYGMSVGFDRKVGDDIIVGGYLTYADSTLTNNSISQDSDNLQL
CapD2_jejuni            WANVIGGANIIDSKSGALYGVSVGYDRLVGEDTILGAYLTYADSKIKTSMVNQESDNLQL
                        *.* :** *:: ...  :** ::* *:   :  : * *:*** :  . . : *.::* *:

CapC1_jejuni            GMYSTINIAPQWELNLKAYAQVSPTKQDNVQVD--GAYNSDYTSKFLGLSANAGRVFDLS
CapC3_jejuni            GMYSTINIAPQWELNLKAYAQVSPTKQDNVQVD--GAYNSDYTSKFLGLSANAGRVFDLS
CapD1_jejuni            GLYSRT-LYGNSEFDFKGYAQFGWTDQDRFIAG--TVNSSDFTRKFLGASGTYGYVFDMG
CapD3_coli              GLYSRT-LYNNSEFDFKTYAQFGWTDQNRLIAQ--TINSSDFTRKFLGASGSYGYVFDIG
CapC4_coli              GMYSTINVAPQWELNLKAYAQVSPTKQDNVQID--GAYNSDYTSKFLGLSANAGRVFDFS
CapC2_coli              GMYSTINVAPQWELNLKAYAQVSPTKQDNVQID--GAYNSDYTSKFLGLSANAGRVFDFS
CapC_upsaliensis        GTYSNIYVTPKVEVNVKAYAQFSPTDQDIVNRGFNTTNSSDFTRKFFGLSANVGYVFDFS
CapC1_lari              GIYSLINPNDQWEINLRAYGQISPTDQSVVMLS--DYSNADFDSKFFGLSANAGRIFNPN
CapC2_lari              GAYSNIYISPKIEVNVKAYAQISPTDQDIINRGFNTTNSADFNRKFFGLSANMGYVFDFS
CapC_subantarcticus     GIYSLINPNDQWEINLRAYGQISPTDQSVMMLS--DSSNANFDSKFFGLSANAGRIFNPN
CapD_subantarcticus     GAYSRI-ANGQHEFDIKTYAQFSWTDQERFLNG--TSNKSDYTQTFLGASGAYGYVFDFS
CapC_peloridis          GAYSNIYITPKVEVNVKAYAQISPTNQDIINRGFNTINSADFNRKFFGLNANVGYVFDFS
CapC_ornithocola        GAYSNIYISPKVEVNVKAYAQISPTDQDIINRGFNTTNSADFNRKFFGLSANMGYVFDFS
CapD_ornithocola        GAYSRI-TNGQHEFDIKTYAQFSFADQERFLNG--TSNKSDFTQTYLGASGSYGYVFDFS
CapD_volucris           GAYSRI-TNGQHEFDIKTYAQFSYTDQERFLNG--TNNKSDYTQTFLGASGAYGYVLDMG
CapC_insulaenigrae      GAYSNIYISPKVEVNVKAYAQFSPTDQDIVNRGFNTTNSADFNRKFFGLSANMGYVFDFS
CapC_cuniculorum        GLYSSIQVAPLWELNLKAYGQVSPTDQSRWDVL--GNYDSDFTRKSFGLSANIGRSFGFQ
CapD4_jejuni            GLYSRI-ATGSHEFDIKGYTQFSFADQNRMIND--SVQTSDFTQTFLGLSGAYGYVFNLN
CapD2_jejuni            GLYSRT-LYGNSEFDFKGYAQFGWTDQDRFIAG--TVNSSDFTRKFLGASGTYGYVFDMG
                        * **        *.:.: * *.. :.*.           :::  . :* ..  *  :.

CapC1_jejuni            DNTLFIKPFAGVNYYFSYTPSHTENGAIAKDIDSMKNNSVSVEVGAEFRKYMNENSYIFV
CapC3_jejuni            DNTLFIKPFAGVNYYFSYTPSHTENGAIAKDIDSMKNNSVSVEVGAEFRKYMNENSYIFV
CapD1_jejuni            ND-FYIKPLAGLNLYYSFTPDYNENGAYAQHVQSQSSFDTSIEAGAEFRKYLSKESYIYA
CapD3_coli              ND-LYIKPLAGLNLYYSLTPSYNENGPYAQHVRSQSNFDASIEAGAEFRKYLSKESYIYA
CapC4_coli              DNTLFIKPFAGVNYYFSYTPSHTENGAIAKDIDSMKNNSVSVEVGAEFRKYMNENSYIFV
CapC2_coli              DNTLFIKPFAGVNYYFSYTPSHTENGAIAKDIDSMKNNSVSVEVGAEFRKYMNENSYIFV
CapC_upsaliensis        DNTLFVKPFTGANYYYAHTPSYKENGVAGKDVKSVSNNSVSLKFGAELRKYMSEESYLFI
CapC1_lari              SSSLFIKPFAGINYYYAHTPSYKESGMFAKEVQSMTNNSISLELGTEFRKYMSETSYLFI
CapC2_lari              DNTLFIKPFAGANYYYAHTPSYKENGIAGKDVSSASNNSISLELGAEFRKYMNEKSYLFI
CapC_subantarcticus     SSSLFIKPFAGINYYYTHTPSYKESGMFAKDVQSMTNNSISLELGAEFRKYMSETSYLFI
CapD_subantarcticus     DN-FSIKPLIGLNLYYGKTPDYTESGVWAQKVYSMDSFAASAELGAEFRKFFNDGSYFYI
CapC_peloridis          NNTLFIKPFAGTNYYYGHTPSYKENGIAGKDVNSASNNSISLELGAEFRKYMSETSYLFI
CapC_ornithocola        DNTLFIKPFAGANYYYAHTPNYSESGVAGKDVNSASNNSISLELGTEFRKYMSEESYLFI
CapD_ornithocola        DN-FSIKPLIGLNLYYSHTPDYTENGLWAQKVYSMDSFAASAELGAEFRKFFNGGSYFYV
CapD_volucris           DK-FAIKPLIGLNLYYGKTPDYTENGVWAQKVYSMDSFAASAEIGAEFRKGFDGGSYFYI
CapC_insulaenigrae      DNTLFVKPFTGANYYYAHTPSYKENGIAGKDVSSASNNSISLEFGAELRKYMSEESYLFI
CapC_cuniculorum        EDTFIVKPFVGANYYLTLTPDYKESGAAGLNIDSSTNNTLSLDLGAEFRKYFNEESYFFI
CapD4_jejuni            NG-FAIKPLAGLNLYYSHTPDYTEEGLWAQHVNSMDSFAFSGDLGVEFRKFFDGGSYIYI
CapD2_jejuni            ND-FYIKPLAGLNLYYSFTPDYNENGAYAQHVQSQSSFDTSIEAGAEFRKYLSKESYIYA
                        .  : :**: * * *   **.:.*.*  . .: *  .   * . *.*:** :.  **::

CapC1_jejuni            TPKIEQFVINSGDDYTANLAVNNAFFTSVEANNKKKTYGQIIVGGNVDFTNQLSMNLGFG
CapC3_jejuni            TPKIEQFVINSGDDYTANLAVNNAFFTSVEANNKKKTYGQIIVGGNVDFTNQLSMNLGFG
CapD1_jejuni            TPKIEQYIITSGDDYTARFLGSPTSFT-INGSDKKKTYGSFIVGGDVNIKNQWAFTFSAG
CapD3_coli              TPKIEQYIITSGDHYTARFLGSPTSFT-INGSDKKKTYGSLIVGGDVNIKNAWAFTFSAG
CapC4_coli              TPKIEQFVINSGDDYTANLAVNNAFFTSVEANNKKKTYGQIIVGGNVDFTNQLSMNLGFG
CapC2_coli              TPKIEQFVINSGDDYTANLAVNNAFFTSVEANNKKKTYGQIIVGGNVDFTNQLSMNLGFG
CapC_upsaliensis        TPKIEQYVINNGDDFVASLNG-VALPS-VKGDDKKKTYGQIIVGGNMDINEQFSLNAGVG
CapC1_lari              TPKIEQYVMNNGDDYVAGFVGSSSNFI-IKGNDKKKTYGQIIVGGNVDINEQFSLNAGIG
CapC2_lari              TPKIEQYVMNNGDDFVASLNG-VALPS-VKSDDKKKTYGQIIIGGNVDISEQFSLNAGIG
CapC_subantarcticus     TPKIEQYVMNNGDDYVAGFVGSNSNFI-IKGNDKKKTYGQIIIGGNVDINEQFSLNAGVG
CapD_subantarcticus     TPKIEQFFAMSGNNFKARFTGSDMNYH-VAGAEKDKTFGKILIGSNIGITDKFTIDLSVG
CapC_peloridis          TPKIEQYVMNNGDDYVASLNG-VALPS-VKSNDKKKTYGQIIIGGNVDINEQFSLNAGIG
CapC_ornithocola        TPKIEQYVMNNGDDFVASLNGSSSTTS-VKSNDKKKTYGQIIVGGNVDINEQFSLNAGIG
CapD_ornithocola        TPKIEQFFAMSGDNFKARFTGSDFSYN-VAGAEKDKTFGKILIGSNISVTDRFSIDLSVG
```

```
CapD_volucris            TPKIEQFFATSGNDFKGRFAGSDMNYH-VSGAEKDKTFGKLLIGSNIGITENLSVDLSLG
CapC_insulaenigrae       TPKIEQYVINNGDDFVASLNG-VTLPS-VKGNDKKKTYGQIIVGGNVDISEQFSLNAGIG
CapC_cuniculorum         TPKVEQYVLNNGGDFEAALAG-IALPS-VDGADKNKTYGQVIIGGNFNLSKQFSANIGLG
CapD4_jejuni             TPKIEQFFVVDGDVFRSGFTGSNLKYS-IHGDESNKTYGQIILGSDISVSKNFSINVSAG
CapD2_jejuni             TPKIEQYIITSGDDYTARFLGSPTSFT-INGSDKKKTYGSFIVGGDVNIKNQWAFTFSAG
                         ***:**:.  .*. : . :         : . :..**:*..::*.:.....  :   . *

CapC1_jejuni             AKQILAGKVDNKNETYLSGQVGLKYKF
CapC3_jejuni             AKQILAGKVDNKNETYLSGQVGLKYKF
CapD1_jejuni             VKHLLSGKVNDESETYLSGNIGLKYQF
CapD3_coli               IKHLLSGKANNESETYLSGNIGLKYQF
CapC4_coli               AKQILAGKVDNKNETYLSGQVGLKYKF
CapC2_coli               AKQILAGKVDNKNETYLSGQVGLKYKF
CapC_upsaliensis         AKQILAGKTDIKNETYLSGQVGFKYKF
CapC1_lari               AKQILAGKTDGKNETYVSGQVGFKYKF
CapC2_lari               AKQILAGKTDGKNETYVSGQVGFKYKF
CapC_subantarcticus      AKQILAGKTDGKNETYVSGQVGFKYKF
CapD_subantarcticus      AKQILGNKDDNTDETYLTGNIGLKYSF
CapC_peloridis           AKQILAGKTDNKNETYVSGQVGFKYKF
CapC_ornithocola         AKQILAGKTDGKNETYVSGQVGFKYKF
CapD_ornithocola         AKQILGNKDDNTDETYITGNIGVKYSF
CapD_volucris            AKQILGNKDDNTDETYFTGNIGFKYSF
CapC_insulaenigrae       AKQILAGKTDGKNETYISGQVGFKYKF
CapC_cuniculorum         VKQILAGKVDSKNETYATGQLGFKYKF
CapD4_jejuni             IKQILGNKDSNTDETYVSGNLGATYKF
CapD2_jejuni             VKHLLSGKVNDESETYLSGNIGLKYQF
                          *::*..* .  .*** :*::* .*.*
```
