# Supplementary figures and images for: Campylobacter jejuni and Campylobacter coli autotransporter genes exhibit lineage-associated distribution and decay
Source: BMC Genomics. 2020 Apr 19;21:314. doi: 10.1186/s12864-020-6704-z (PMC7168839; doi:10.1186/s12864-020-6704-z)

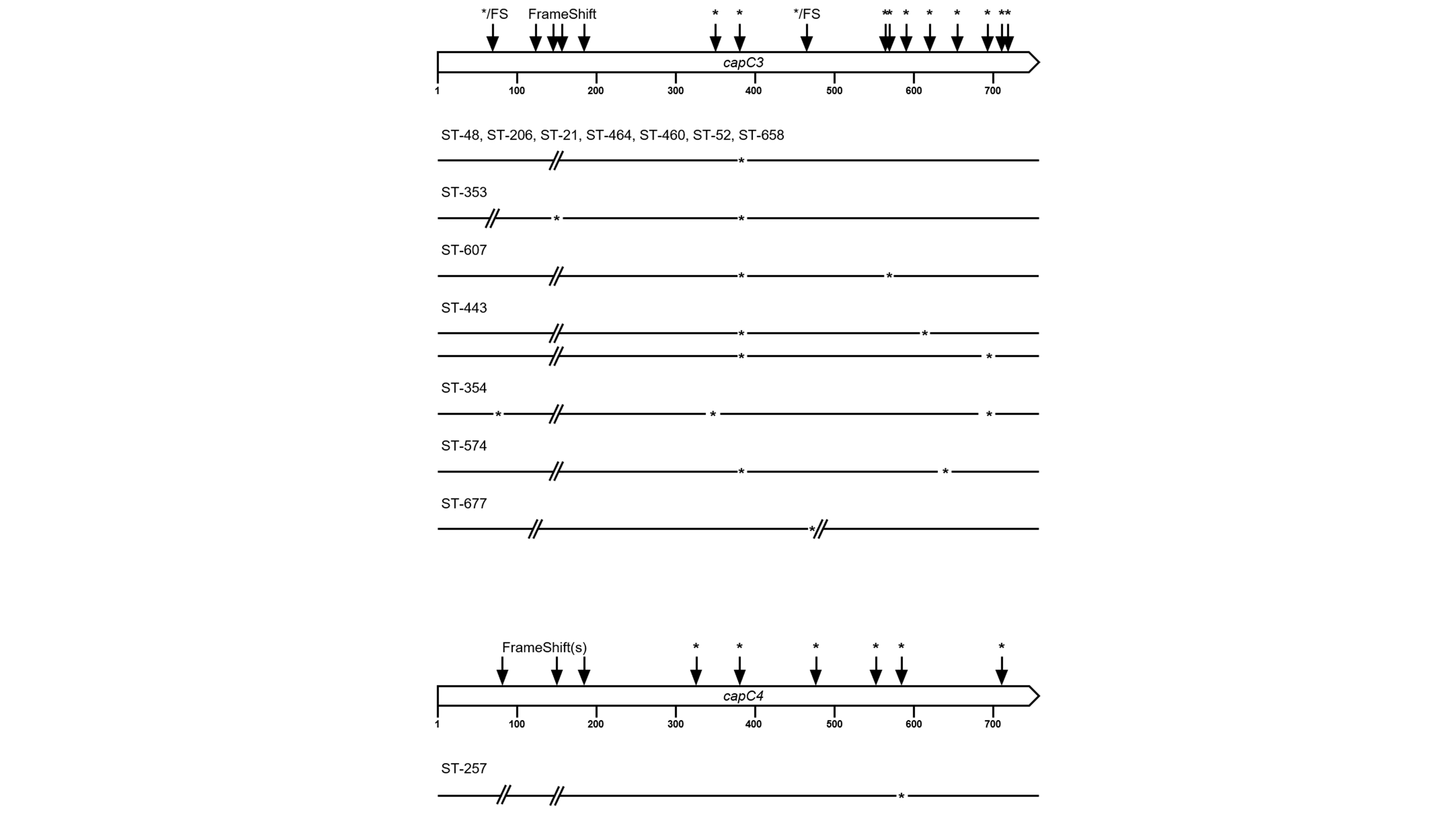

Supplement: Supplementary file 3 — Additional file 3. Figure displaying the fragmentation patterns of inactive capC3 and capC4 genes. The figure shows the various frameshifts (FS) and point mutations that result in inactive genes; these mutations are associated with the clonal complex in which the inactive capC3/capC4 is present. [file 12864_2020_6704_MOESM3_ESM.tif]
